# Agelasine Diterpenoids and Cbl-b Inhibitory Ageliferins from the Coralline Demosponge *Astrosclera willeyana*

**DOI:** 10.3390/md19070361

**Published:** 2021-06-24

**Authors:** Wei Jiang, Dongdong Wang, Brice A. P. Wilson, Unwoo Kang, Heidi R. Bokesch, Emily A. Smith, Antony Wamiru, Ekaterina I. Goncharova, Donna Voeller, Stanley Lipkowitz, Barry R. O’Keefe, Kirk R. Gustafson

**Affiliations:** 1 Marine Science & Technology Institute, College of Environmental Science & Engineering, Yangzhou University, Yangzhou 225127, China; weijiang@yzu.edu.cn; 2 Molecular Targets Program, Center for Cancer Research, National Cancer Institute, National Institutes of Health, Frederick, MD 21702-1201, USA; dongdong.wang@nih.gov (D.W.); brice.wilson@nih.gov (B.A.P.W.); smileunu@gmail.com (U.K.); heidibokesch@hotmail.com (H.R.B.); emily.smith2@nih.gov (E.A.S.); wamiruam@mail.nih.gov (A.W.); goncharovae@mail.nih.gov (E.I.G.); okeefeba@mail.nih.gov (B.R.O.); 3 Basic Science Program, Frederick National Laboratory for Cancer Research, National Institutes of Health, Frederick, MD 21702-1201, USA; 4 Advanced Biomedical Computational Science, Frederick National Laboratory for Cancer Research, Frederick, MD 21702-1201, USA; 5 Women’s Malignancies Branch, Center for Cancer Research, National Cancer Institute, National Institutes of Health, Bethesda, MD 20892-1578, USA; donna.voeller@nih.gov (D.V.); lipkowis@navmed.nci.nih.gov (S.L.); 6 Natural Products Branch, Developmental Therapeutics Program, Division of Cancer Treatment and Diagnosis, National Cancer Institute, National Institutes of Health, Frederick, MD 21702-1201, USA

**Keywords:** ageliferins, agelasine diterpenoids, *Astrosclera willeyana*, Cbl-b inhibition, *N*-methyladenine, bromopyrrole

## Abstract

An extract of the coralline demosponge *Astrosclera willeyana* inhibited the ubiquitin ligase activity of the immunomodulatory protein Cbl-b. The bioassay-guided separation of the extract provided ten active compounds, including three new *N*-methyladenine-containing diterpenoids, agelasines W–Y (**1**–**3**), a new bromopyrrole alkaloid, *N*(1)-methylisoageliferin (**4**), and six known ageliferin derivatives (**5**–**10**). The structures of the new compounds were elucidated from their spectroscopic and spectrometric data, including IR, HRESIMS, and NMR, and by comparison with spectroscopic data in the literature. While all of the isolated compounds showed Cbl-b inhibitory activities, ageliferins (**4**–**10**) were the most potent metabolites, with IC_50_ values that ranged from 18 to 35 μM.

## 1. Introduction

The ubiquitin protein ligase (E3), referred to as Casitas B-lineage lymphoma proto-oncogene-b (Cbl-b), negatively regulates the costimulatory pathway in T cells, decreasing the immune response and setting the threshold for anergy in T cells [1]. Cbl-b is essential for the negative regulation of T-cell activation, and thus, it reduces the immune response to cancer cells [2,3]. In line with this function, cells that lack the *cblb* gene rejected tumors in various models and were resistant to rechallenge with tumors after initial tumor rejection in a variety of tumor models [4,5,6,7]. Thus, targeting Cbl-b may be an effective strategy to enhance antitumor immunity. As part of an ongoing effort to identify small molecule inhibitors of the Cbl-b function from natural products [8], an extract of the sponge *Astrosclera willeyana* was screened and showed a marked reduction of Cbl-b ligase activity.

The calcareous demosponge *Astrosclera willeyana* is often referred to as a “living fossil” that is representative of late Paleozoic and Mesozoic reef sponges, and it has provided unique secondary metabolites such as *N*-methylated ageliferin derivatives and manzacidin D [9,10]. These compounds contain pyrrole-2-carboxylic acid moieties, and their novel structures have been the focus of numerous synthetic efforts [11,12,13,14,15]. To date, little is known about the biological properties of these compounds, except for the reported cytotoxic and antibacterial activities of the nonmethylated form of ageliferin [16,17]. In our current study, bioassay-guided fractionation of the *A. willeyana* extract provided ten active compounds, including three new *N*-methyladenine-containing diterpeneoids named agelasines W–Y (**1**–**3**). The agelasines represent a family of diterpene–adenine conjugates that has only been described from sponges in the genus *Agelas*. In addition, a new bromopyrrole alkaloid, *N*(1)-methylisoageliferin (**4**), along with six known ageliferin derivatives (**5**–**10**) were isolated and identified. Herein, we describe the isolation, structure elucidation, and biological activities of the *A. willeyana* metabolites.

## 2. Results and Discussion

**Figure 1:**
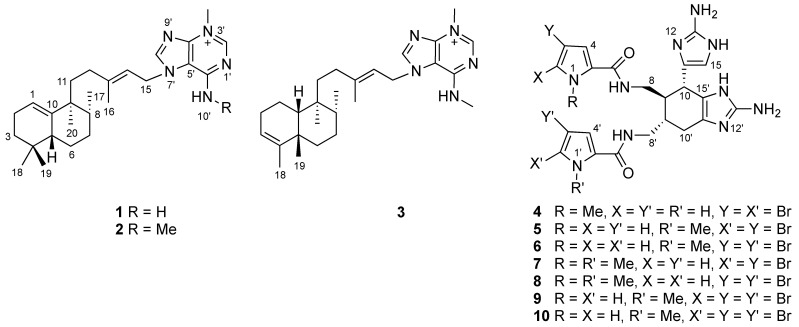
Structures of compounds **1**–**10**.

The organic solvent extract of the sponge *Astrosclera willeyana* was separated by bioassay-guided diol flash chromatography and C_18_ HPLC to yield four new metabolites named agelasines W–Y (**1**–**3**) and *N*(1)-methylisoageliferin (**4**) (Figure 1), along with six known compounds: *N*(1′)-methylisoageliferin (**5**), *N*(1′)-methylageliferin (**6**), *N*(1),*N*(1′)-dimethylisoageliferin (**7**), *N*(1),*N*(1′)-dimethylageliferin (**8**), *N*(1′)-methyl-2-bromoageliferin (**9**), and *N*(1′)-methyl-2′-bromoageliferin (**10**).

**Figure 2:**
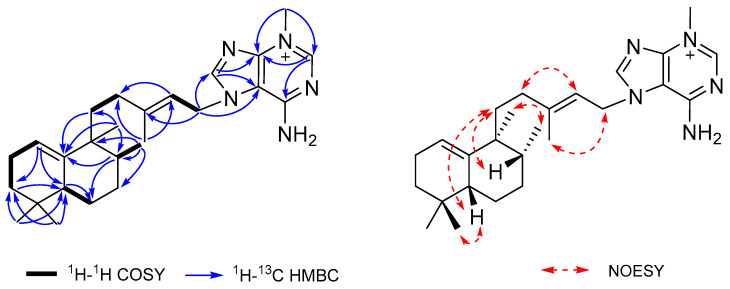
Key 2D correlations for agelasine W (**1**).

Agelasine W (**1**) was obtained as a colorless oil. The molecular formula C_26_H_40_N_5_^+^, with 10 degrees of unsaturation, was determined by HRESIMS measurements ([M]^+^ *m*/*z* 422.3285, calcd for C_26_H_40_N_5_^+^, 422.3278). The ^1^H and ^13^C NMR data (Table 1) of compound **1** showed characteristic signals of an adenine moiety at *δ*_H_/*δ*_C_ 8.44 (1H, s, H-8′)/148.0 (C-8′), 8.57 (1H, s, H-2′)/149.5 (C-2′), *δ*_C_ 112.4 (C-5′), 151.5 (C-4′), and 155.1 (C-6′), and an *N*-methyl group at *δ*_H_/*δ*_C_ 4.04 (3H, s)/36.6, revealing the presence of an *N*-methyladeninium unit in (**1**). The remaining C_20_H_33_ portion was defined as a halimane diterpenoid moiety by comparison of its NMR spectroscopic data with those of related diterpenes [18,19,20]. The four methyl singlets at *δ*_H_ 0.84 (H_3_-18), 0.88 (H_3_-19), 0.94 (H_3_-20), and 1.84 (H_3_-16), and a doublet at *δ*_H_ 0.83 (3H, d, *J* = 6.4 Hz, H-17), were compatible with a bicyclic halimane ring system, while the ^1^H NMR signals of *δ*_H_ 2.10 (1H, m, H-11a), 1.26 (1H, m, H-11b), 2.00 (1H, m, H-12a), 1.81 (1H, m, H-12b), 5.45 (1H, t, *J* = 6.9 Hz, H-14), 5.12 (2H, br d, *J* = 6.9 Hz, H_2_-15), and 1.84 (3H, s, H_3_-16) were assigned to a 3-methyl-2-pentenyl chain, according to their COSY and HMBC correlations (Figure 2). The HMBC correlations from H-1 to C-3 and C-5, and from H-8 and H_2_-11 to C-10, established the location of a trisubstituted olefin at Δ^1−10^, while the HMBC correlations from H_2_-15 to C-5′ and C-8′ defined the attachment of C-15 to N-7′ of the adenine subunit. Moreover, the ^1^H and ^13^C data of (**1**) were highly similar to those of agelasine C [21]. However, the *N*-methyl group in (**1**) showed HMBC correlations to C-2′ and C-4′, which revealed its location on N-3′, while agelasine C had a methyl group substituted at N-9′. The *E* configuration of the C-13/C-14 olefin was assigned from NOESY correlations between H-14/H-12b and H_2_-15/H_3_-16. Additional NOESY correlations of H-5/H-11a and H-8/H-11b suggested that H-5, H-8, and H_2_-11 were on the same face of the molecule, while a correlation between H_3_-17 and H_3_-20 supported this assignment. The chemical shift of C-20 appeared at *δ*_H_ 0.94 ppm, which was consistent with the C-17 and C-20 methyl groups being *cis,* since it was reported that C-20 is more shielded in a *cis* than a *trans* orientation of these methyls [21]. The absolute configuration of agelasine W (**1**) is suggested as 5*R*, 8*R*, 9,*S* according to the positive optical rotation of (**1**) ([*α*]${}_{D}^{25}$ + 17) compared to those for (+)-and (−)-agelasine C, +36.7 and −55.1, respectively [20,21].

Agelasine X (**2**) was obtained as a colorless oil, and the HRESIMS spectrum displayed a [M]^+^ ion at *m*/*z* 436.3452, corresponding to the molecular formula of C_27_H_42_N_5_^+^ with 10 degrees of unsaturation. The ^1^H and ^13^C NMR data of compound **2** were nearly identical to those of (**1**), except for the presence of an additional *N*-methyl group at *δ*_H_/*δ*_C_ 3.27 (3H, s)/29.3. The location of the *N*-methyl group was assigned at N-10′ based on an HMBC correlation to C-6′ (*δ*_C_ 153.8). The relative and absolute configurations of agelasine X (**2**) were assigned the same as (**1**) based on their close spectroscopic similarities and its positive optical rotation ([*α*]${}_{D}^{25}$ + 20).

**Figure 3:**
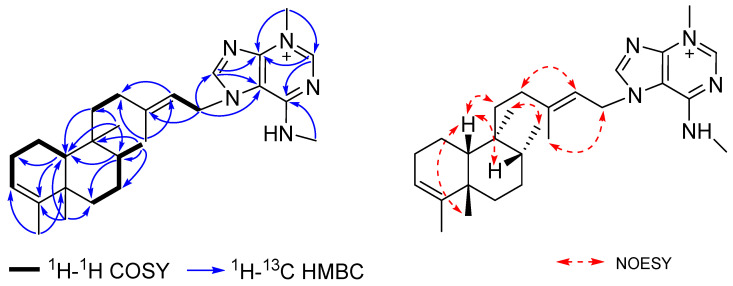
Key 2D NMR correlations for agelasine Y (**3**).

Agelasine Y (**3**) was also isolated as a colorless oil, and HRESIMS ([M]^+^ *m*/*z* 436.3439, calcd for C_27_H_42_N_5_^+^, 436.3435) established a molecular formula of C_27_H_42_N_5_^+^ that was isomeric with compound **2**. Agelasine Y (**3**) shared many similar NMR features with those of (**2**), except for differences in select signals in the bicyclic diterpene ring system (Table 1). The diterpene portion of (**3**) was assigned as a clerodane skeleton by NMR analysis and a comparison of its spectroscopic data with those of previously reported clerodane diterpenes [20,22,23,24]. The location of the trisubstituted C-3/C-4 olefin was defined by a COSY correlation between the H-2/H-3 and HMBC correlations from H_2_-1 to C-3, H-10 to C-4, H_3_-18 to C-3 and C-5, and from H_3_-19 to C-4 (Figure 3). The relative configuration of the bicyclic ring system of (**3**) was deduced as a *cis*-clerodane from the characteristic deshielded carbon signals at *δ*_C_ 25.0 (CH_2_, C-2) and 33.6 (CH_3_, C-19) [22], in addition to a NOESY correlation between H-10 and H_3_-19. Additional NOESY correlations of H-8/H-10, H-10/H-11a, and H_3_-17/H_3_-20 established the relative configurations at C-8 and C-9. Furthermore, a comparison of the ^13^C NMR data in CDCl_3_ with four closely related clerodane diastereomers (*cis-cis*, *cis-trans*, *trans-trans*, and *trans-cis*) showed that the chemical shifts of (**3**) (Supplementary Materials) were in good agreement with those of *neo-cis-cis*-kolavenol [25], supporting the assigned configuration of the bicyclic scaffold. The *E* configuration of the side chain olefin was assigned from NOESY correlations of H-14/H-12b and H_2_-15/H_3_-16. The structure of (**3**) resembled that of agelasine Except for the position of the *N*-methyl groups, the rotation for (**3**) ([*α*]${}_{D}^{25}$ + 15) and agelasine A ([*α*]${}_{D}^{25}$ − 31.3) have opposite signs [20]. Therefore, the absolute configuration of agelasine Y (**3**) is suggested as shown.

**Figure 4:**
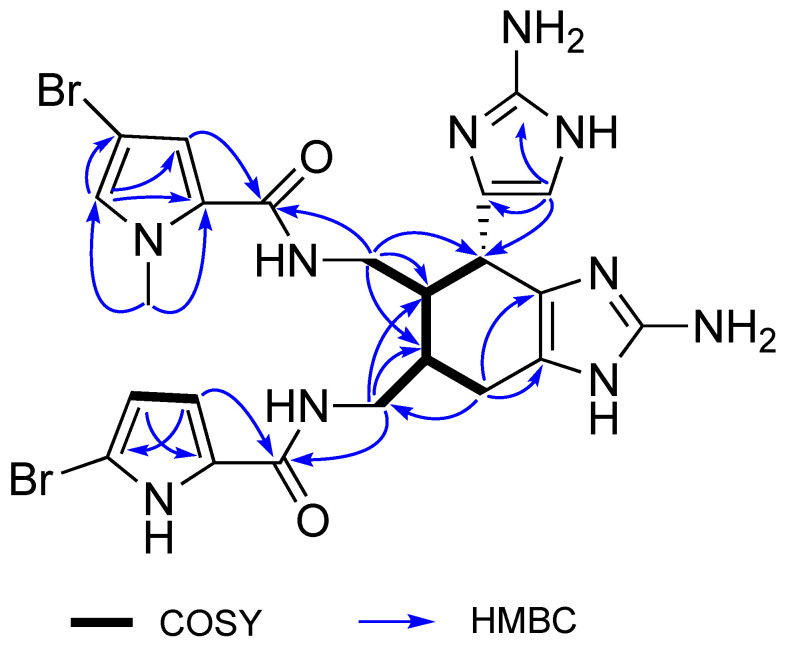
Key 2D NMR correlations for *N*(1)-methylisoageliferin (**4**).

*N*(1)-methylisoageliferin (**4**) was obtained as a pale-yellow glass. The molecular formula C_23_H_26_Br_2_N_10_O_2_ with 15 degrees of unsaturation was determined by an HRESIMS of the doubly charged ion ([M + 2H]^2+^ *m*/*z* 317.0374, calcd for C_23_H_28_N_10_O_2_^79^Br_2_^2+^, 317.0376). The ^1^H and ^13^C NMR data of (**4**) (Table 2) closely resembled those of the known compound *N*(1′)-methylisoageliferin (**5**) [10], and the molecular formula of (**4**) was isomeric with (**5**). NMR signals characteristic of the 3-bromo-*N*-methylpyrrole 5-carbozamide and 2-bromopyrrole 5-carboxamide ring systems were apparent, as well as signals for a highly substituted cyclohexene and two amino imidazole rings. The HMBC correlations from H-4 and H_2_-8 to C-6 (Figure 4) revealed that the 3-bromo*-N*-methylpyrrole 5-carboxamide ring was linked to C-8, and the 2-bromopyrrole 5-carboxamide ring was linked to C-8′ via the HMBC correlations from H-4′ and H_2_-8′ to C-6′. Thus, the constitution of (**4**) only differed from (**5**) by the location of *N-*methyl substitution on the pyrrole ring. The configuration of *N*(1)-methylisoageliferin (**4**) was identical to that of (**5**), since its NMR data for the cyclohexene ring, including proton coupling constants and NOE enhancements, and its optical rotation, as well as its ECD data, were fully consistent with those of (**5**) [10].

The known compounds **5**–**10** were identified as *N*(1′)-methylisoageliferin (**5**), *N*(1′)-methylageliferin (**6**), *N*(1),*N*(1′)-dimethylisoageliferin (**7**), *N*(1),*N*(1′)-dimethylageliferin (**8**), *N*(1′)-methyl-2-bromoageliferin (**9**), and *N*(1′)-methyl-2′-bromoageliferin (**10**) by comparison of their spectroscopic data with the appropriate literature values [10].

Compounds **1**–**10** were tested for their ability to inhibit the in vitro enzymatic activity of the Cbl-b ubiquitin ligase (Table 3). The adenine–diterpenoid metabolites agelasines W–Y (**1**–**3**) showed weak inhibitory activities against Cbl-b (IC_50_ > 50 µM), while the ageliferin derivatives (**4**–**10**) had more pronounced inhibitory effects (IC_50_ = 18~35 µM). Compounds **7**–**9** were the most potent metabolites among the tested compounds, but definitive SAR conclusions were difficult to make. There was no clear pattern of *N-*methylation or bromine substitution of the two pyrrole rings that directly correlated with the observed Cbl-b inhibitory activity. While the ageliferins are rather modest inhibitors of Cbl-b, the ageliferin structural scaffold could serve as a starting point for the development of more potent analogs with enhanced inhibitory properties.

## 3. Materials and Methods

### 3.1. General Experimental Procedures

Flash chromatography was performed using a CombiFlash system (Teledyne Isco, Lincoln, NE, USA). High-performance liquid chromatography (HPLC) was performed using a Varian ProStar 215 solvent delivery module equipped with a Varian ProStar 340 UV-Vis detector, operating under Star 6.41 chromatography workstation software (Agilent Technologies, Santa Clara, CA, USA). HPLC fractions were subsequently dried on Explorer-220 (Thermo Fisher Scientific, Waltham, MA, USA). NMR spectra were acquired with a Bruker Avance III NMR spectrometer equipped with a 3-mm cryogenic probe and operated at 600 MHz for ^1^H and 150 MHz for ^13^C (Bruker, Billerica, MA, USA). Spectra were calibrated to their residual solvent signals at *δ*_H_ 3.31 and *δ*_C_ 49.0 for CD_3_OD. LRESIMS studies were measured on an Agilent 6130 Quadrapole LC/MS system (Agilent Technologies, Santa Clara, CA, USA). HRESIMS data were carried out on an Agilent Technology 6530 Accurate-mass Q-TOF LC/MS (Agilent Technologies, Santa Clara, CA, USA). UV spectra were measured with a PerkinElmer Lambda 465 UV/Vis photodiode array spectrophotometer (PerkinElmer, Waltham, MA, USA). ECD spectra were obtained with a Jasco-1500 circular dichroism spectrophotometer (JASCO, Tokyo, Japan). Optical rotations were recorded on a Rudolph research analytical AUTOPOL IV spectropolarimeter (Rudolph Research Analytical, Hackettstown, NJ, USA). IR spectra were measured with a Bruker ALPHA II FT-IR spectrometer (Bruker, Billerica, MA, USA).

### 3.2. Animal Material

Specimens of the sponge *Astrosclera willeyana* were collected in Tonga in November 1997 and kept frozen until extraction. The collection was carried out by the Coral Reef Research Foundation under contract with the Natural Products Branch, U.S. National Cancer Institute. A voucher specimen (voucher ID # 0CDN5435) was deposited at the Smithsonian Institution, Washington, DC, USA.

### 3.3. Extraction and Isolation

Following the standard NCI protocol for marine samples [26], the frozen sponge sample (2127 g, wet weight) was ground and processed to provide 4.50 g of organic solvent (CH_2_Cl_2_-MeOH 1:1 and 100% MeOH) extract (NSC #C017821). A 3.10-g aliquot of the extract was loaded onto a cotton plug and then applied to a diol flash chromatography column (150 g) through a CombiFlash system, sequentially eluting with hexane, CH_2_Cl_2_, EtOAc/CH_2_Cl_2_ 1:1 (*v*/*v*), EtOAc, MeOH/EtOAc 1:4 (*v*/*v*), MeOH/EtOAc 1:1 (*v*/*v*), MeOH/EtOAc 4:1 (*v*/*v*), and MeOH to obtain nine fractions (Frac. A–I). The active Frac. E (eluted by MeOH/EtOAc 1:4, (*v*/*v*) 735 mg) was fractionated by semipreparative HPLC (Phenomenex Luna C18(2), 5 µM, 100 Å, 250 × 21.2 mm), using a linear gradient of CH_3_CN/H_2_O 3:7–1:0 with 0.1% trifluoroacetic acid (TFA) as the mobile phase to afford agelasine W (**1**, 7.0 mg), agelasine X (**2**, 18.9 mg), and agelasine Y (**3**, 4.7 mg). Another more active Frac. F (eluted by EtOAc/MeOH 1:1, 1787 mg) was separated by semipreparative HPLC in the same way as Frac. E to afford *N*(1)-methylisoageliferin (**4**, 10.7 mg), *N*(1′)-methylisoageliferin (**5**, 20.0 mg), *N*(1′)-methylageliferin (**6**, 26.3 mg), *N*(1),*N*(1′)-dimethylisoageliferin (**7**, 53.1 mg), *N*(1), *N*(1′)-dimethylageliferin (**8**, 6.9 mg), *N*(1′)-methyl-2-bromoageliferin (**9**, 26.9 mg), and *N*(1′)-methyl-2′-bromoageliferin (**10**, 14.7 mg).

*Agelasine W* (**1**): colorless oil; [*α*]${}_{D}^{25}$ + 17 (*c* 0.06, MeOH); UV (MeOH) *λ*_max_ (log *ε*) 280 (3.70) nm; IR (neat) *ν*_max_ 2951, 2357, 1659, 1434, 1196, 1133 cm^−1^; ^1^H and ^13^C NMR, Table 1; HRESIMS *m*/*z* 422.3285 [M]^+^ (calcd for C_26_H_40_N_5_^+^, 422.3278).

*Agelasine X* (**2**): colorless oil; [*α*]${}_{D}^{25}$ + 20 (*c* 0.06, MeOH); UV (MeOH) *λ*_max_ (log *ε*) 285 (3.89); IR (neat) *ν*_max_ 2952, 2357, 1643, 1395, 1196, 1132 cm^−1^; ^1^H and ^13^C NMR, Table 1; HRESIMS *m*/*z* 436.3452 [M]^+^ (calcd for C_27_H_42_N_5_^+^, 436.3435).

*Agelasine Y* (**3**): colorless oil; [*α*]${}_{D}^{25}$ + 15 (*c* 0.06, MeOH); UV (MeOH) *λ*_max_ (log *ε*) 285 (3.95) nm; IR (neat) *ν*_max_ 2944, 2357, 1643, 1394, 1196, 1132 cm^−1^; ^1^H and ^13^C NMR, Table 1; HRESIMS *m*/*z* 436.3439 [M]^+^ (calcd for C_27_H_42_N_5_^+^, 436.3435).

*N(1)-Methylisoageliferin* (**4**): pale-yellow glass; [*α*]${}_{D}^{25}$ + 50 (*c* 0.06, MeOH); UV (MeOH) *λ*_max_ (log *ε*) 218 (4.30), 228 (4.25) and 270 (4.32) nm; ECD (*c* 7.12 × 10^−3^ M, MeOH) *λ*_max_ (Δ*ε*) 284 (−0.75), 250 (+0.96), 229 (−4.56), and 212 (+1.33) nm; IR (neat) *ν*_max_ 3172, 1678, 1200, 1139 cm^−1^; ^1^H and ^13^C NMR, Table 2; HRESIMS *m*/*z* 317.0374 [M + 2H]^2+^ (calcd for C_23_H_28_N_10_O_2_^79^Br_2_^2+^, 317.0376).

### 3.4. Cbl-b Biochemical Assay

An extract of *Astrosclera willeyana* was identified as an active source in a screening campaign of prefractionated natural product samples for the inhibition of Cbl-b ubiquitin ligase activity [8]. The bulk extract was fractionated as described above, and the resulting fractions were evaluated for activity in the Cbl-b bioassay, the details of which have already been reported [8]. In brief, dose response experiments with chromatography fractions and purified compounds were carried out in a Tris-HCl-based enzymatic assay buffer with the following final composition: 75-nM Cbl-b (N1/2 construct) [27], 50-nM Ube2d2 protein (E2) [28], 10-nM UBE1protein (E1), 50-nM biotinylated ubiquitin, and 0.5-µM unlabeled recombinant ubiquitin. The assay buffer also contained the following additives: 0.1-mM dithiothreitol, 0.5-mg/mL bovine gelatin (type B), 0.5-mM magnesium chloride, and 0.01% Triton X-100. Reactions were initiated by the addition of ATP to a final concentration of 100 µM. Following initiation, reactions were transferred to plates previously precoated overnight with 10 µg/mL of the polyubiquitin-binding domain of Cbl-b (UBA) [29]. The UBA domain of Cbl-b binds ubiquitin chains, which results in the capture and enrichment of autopolyubiquitinated Cbl-b. Following a reaction interval of 60 min, the reactions were quenched by the addition of zinc to a final concentration of 2.4 mM. Quenched reactions were incubated at room temperature overnight. Following this incubation period reaction, plates were processed as standard ELISA plates: washed thrice in Tris-buffered saline (with 0.1% Tween-20, 1X TBST), probed with avidin-conjugated horse radish peroxidase, which binds captured biotinylated ubiquitin, washed again in 1X TBST (3X), and then, an avidin-HRP-dependent fluorescent signal (indicating avidin-HRP/biotin-polyubiquitin complexes captured by the UBA coating on the plate) was detected (excitation 325 nm, emission 420 nm) using an Infinite M1000 (Tecan US, Inc., Raleigh, NC, USA) plate reader.

## 4. Conclusions

Two different classes of Cbl-b inhibitory metabolites, including three new adenine-diterpenoid conjugates (**1**–**3**) and seven ageliferin derivatives (**4**–**10**), were obtained from the marine sponge *Astrosclera willeyana*. Agelasines W–Y (**1**–**3**) have bicyclic terpenoid skeletons with a prenyl side chain that terminates with an *N*-methyladenine subunit. These alkaloids arise from a mixed biosynthetic process that incorporates both terpene and purine components, and their new structures enrich the known chemo-diversity of *Astrosclera* sponges. Diterpene alkaloids with an *N*-methyladenine moiety are generally methylated at the N-9′ position [30,31,32,33,34]; however, agelasines W–Y (**1**–**3**), along with the previously reported agelasimines [35,36], have methyl substituents at both the N-3′ and N-10′ positions. The new compound *N*(1)-methylisoageliferin (**4**) and six known analogs of ageliferin (**5**–**10**) exhibited significant Cbl-b inhibitory properties, and they could provide a structural framework for lead compound development.

## Figures and Tables

**Table 1:** ^1^H NMR (600 MHz) and ^13^C NMR (150 MHz) data for agelasines W–Y (**1**–**3**) in CD_3_OD.

Position	1		2		3	
	*δ*_H_ (*J* in Hz)	*δ*_C_, Type	*δ*_H_ (*J* in Hz)	*δ*_C_, Type	*δ*_H_ (*J* in Hz)	*δ*_C_, Type
1	5.36, t (4.0)	121.4, CH	5.36, t (4.0)	121.4, CH	2.01, m 1.83, m	18.8, CH_2_
2	2.04, m	24.1, CH_2_	2.04, m	24.1, CH_2_	2.15, m 2.01, m	25.0, CH_2_
3	1.37, m 1.13, m	34.2, CH_2_	1.37, m 1.13, m	34.2, CH_2_	5.28, br s	124.4, CH
4		32.4, C		32.4, C		141.0, C
5	1.69, m	44.8, CH	1.69, m	44.9, CH		38.0, C
6	1.59, m 1.30, m	24.8, CH_2_	1.59, m 1.30, m	24.8, CH_2_	2.03, m 1.09, m	38.8, CH_2_
7	2.02, m 1.37, m	30.2, CH_2_	2.02, m 1.37, m	30.2, CH_2_	1.25, m	29.9, CH_2_
8	1.55, m	40.6, CH	1.55, m	40.6, CH	1.48, m	38.6, CH
9		44.1, C		44.1, C		41.3, C
10		142.7, C		142.6, C	1.40, m	45.9, CH
11	2.10, m 1.26, m	38.6, CH_2_	2.10, m 1.26, m	38.7, CH_2_	1.65, m 1.37, m	37.6, CH_2_
12	2.00, m 1.81, m	35.5, CH_2_	2.00, m 1.81, m	35.5, CH_2_	2.03, m	33.9, CH_2_
13		147.4, C		147.7, C		147.7, C
14	5.45, t (6.9)	117.5, CH	5.45, t (6.9)	117.2, CH	5.50, t (7.0)	117.3, CH
15	5.12, br d (6.9)	46.6, CH_2_	5.13, br d (6.9)	46.7, CH_2_	5.15, br d (7.0)	46.7, CH_2_
16	1.84, s	17.0, CH_3_	1.84, s	17.0, CH_3_	1.86, s	17.0, CH_3_
17	0.83, d (6.4)	16.0, CH_3_	0.83, d (6.4)	16.0, CH_3_	0.80, d (6.4)	16.3, CH_3_
18	0.84, s	26.6, CH_3_	0.84, s	26.6, CH_3_	1.69, s	20.0, CH_3_
19	0.88, s	28.7, CH_3_	0.88, s	28.7, CH_3_	1.04, s	33.6, CH_3_
20	0.94, s	22.8, CH_3_	0.94, s	22.8, CH_3_	0.85, s	17.9, CH_3_
2′	8.57, s	149.5, CH	8.67, s	149.5, CH	8.67, s	149.5, CH
3′-*N*Me	4.04, s	36.6, CH_3_	4.06, s	36.6, CH_3_	4.05, s	36.6, CH_3_
4′		151.5, C		150.0, C		150.4, C
5′		112.4, C		113.1, C		113.1, C
6′		155.1, C		153.8, C		153.8, C
8′	8.44, s	148.0, CH	8.38, s	147.1, CH	8.39, s	147.1, CH
10′-*N*Me			3.27, s	29.3, CH_3_	3.27, s	29.3, CH_3_

**Table 2:** ^1^H NMR (600 MHz) and ^13^C (150 MHz) NMR data for *N*(1)-methylisoageliferin (**4**) in CD_3_OD.

Position	*δ*_H_ (*J* in Hz)	*δ*_C_, Type
2	6.91, d (1.5)	129.1, CH
2′		104.7, C
3		95.6, C
3′	6.14, d (4.0)	112.5, CH
4	6.84, d (1.5)	116.1, CH
4′	6.81, d (4.0)	113.5, CH
5		127.0, C
5′		128.4, C
6		163.9, C
6′		163.0, C
8	3.72, dd (14.8, 3.2); 3.43, dd (14.8, 4.3)	40.4, CH_2_
8′	3.63, dd (14.0, 2.7); 3.37, dd (14.0, 2.7)	42.5, CH_2_
9	2.17, m	43.8, CH
9′	2.25, m	37.2, CH
10	3.82, br d (8.5)	33.6, CH
10′	2.72, dd (16.3, 5.3); 2.47, ddd (16.3, 9.0, 2.9)	23.5, CH_2_
11		127.6, C
11′		122.8, C
13		149.3, C
13′		149.2, C
15	6.77, s	112.9, CH
15′		119.1, C
NMe	3.90, s	37.2, CH_3_

**Table 3:** Cbl-b inhibitory activities of compounds **1**–**10** (IC_50_ values in μM).

Compound	IC_50_	Compound	IC_50_
**1**	57	**6**	30
**2**	72	**7**	18
**3**	66	**8**	19
**4**	33	**9**	19
**5**	25	**10**	35

## Data Availability

The data presented in this study are available in the Supplementary Materials.

## Supplementary Materials

The following are available online at https://www.mdpi.com/article/10.3390/md19070361/s1, Figure S1: ^1^H NMR spectrum (600 MHz) of Agelasine W (**1**) in CD_3_OD, Figure S2: ^13^C NMR spectrum (150 MHz) of Agelasine W (**1**) in CD_3_OD, Figure S3: HSQC spectrum of Agelasine W (**1**) in CD_3_OD, Figure S4: HMBC spectrum of Agelasine W (**1**) in CD_3_OD, Figure S5: COSY spectrum of Agelasine W (**1**) in CD_3_OD, Figure S6: NOESY spectrum of Agelasine W (**1**) in CD_3_OD, Figure S7: HRESIMS of Agelasine W (**1**), Figure S8: IR spectrum of Agelasine W (**1**), Figure S9: UV spectrum of Agelasine W (**1**), Figure S10: ^1^H NMR spectrum (600 MHz) of Agelasine X (**2**) in CD_3_OD, Figure S11: ^13^C NMR spectrum (150 MHz) of Agelasine X (**2**) in CD_3_OD, Figure S12: HSQC spectrum of Agelasine X (**2**) in CD_3_OD, Figure S13: HMBC spectrum of Agelasine X (**2**) in CD_3_OD, Figure S14: COSY spectrum of Agelasine X (**2**) in CD_3_OD, Figure S15: HRESIMS of Agelasine X (**2**), Figure S16: IR spectrum of Agelasine X (**2**), Figure S17: UV spectrum of Agelasine X (**2**), Figure S18: ^1^H NMR spectrum (600 MHz) of Agelasine Y (**3**) in CD_3_OD, Figure S19: ^13^C NMR spectrum (150 MHz) of Agelasine Y (**3**) in CD_3_OD, Figure S20: HSQC spectrum of Agelasine Y (**3**) in CD_3_OD, Figure S21: HMBC spectrum of Agelasine Y (**3**) in CD_3_OD, Figure S22: COSY spectrum of Agelasine Y (**3**) in CD_3_OD, Figure S23: NOESY spectrum of Agelasine Y (**3**) in CD_3_OD, Figure S24: HRESIMS of Agelasine Y (**3**), Figure S25: IR spectrum of Agelasine Y (**3**), Figure S26: UV spectrum of Agelasine Y (**3**), Figure S27: ^1^H NMR spectrum (600 MHz) of *N*(1)-methylisoageliferin (**4**) in CD_3_OD, Figure S28: ^13^C NMR spectrum (150 MHz) of *N*(1)-methylisoageliferin (**4**) in CD_3_OD, Figure S29: HSQC spectrum of *N*(1)-methylisoageliferin (**4**) in CD_3_OD, Figure S30. HMBC spectrum of *N*(1)-methylisoageliferin (**4**) in CD_3_OD, Figure S31: COSY spectrum of *N*(1)-methylisoageliferin (**4**) in CD_3_OD, Figure S32: ECD spectrum of *N*(1)-methylisoageliferin (**4**), Figure S33: HRESIMS of *N*(1)-methylisoageliferin (**4**), Figure S34: IR spectrum of *N*(1)-methylisoageliferin (**4**), Figure S35: UV spectrum of *N*(1)-methylisoageliferin (**4**), Table S1: ^1^H NMR (600 MHz) data for compounds **5**–**10** in CD_3_OD, Table S2: ^13^C NMR (150 MHz) data for compound **5**–**10** in CD_3_OD, Table S3: ^13^C NMR (150 MHz) data for compound **3** and closely related clerodane diastereomers in CDCl_3_.

## References

[1] Lutz-Nicoladoni C., Wolf D., Sopper S. (2015). Modulation of immune cell functions by the E3 ligase Cbl-b. Front. Oncol..

[2] Paolino M., Penninger J.M. (2010). Cbl-b in T-cell activation. Semin. Immunopathol..

[3] Wallner S., Gruber T., Baier G., Wolf D. (2012). Releasing the brake: Targeting Cbl-b to enhance lymphocyte effector functions. Clin. Dev. Immunol..

[4] Bachmaier K., Krawczyk C., Kozieradzki I., Kong Y.-Y., Sasaki T., Oliveira-Dos-Santos A., Mariathasan S., Bouchard D., Wakeham A., Itie A. (2000). Negative regulation of lymphocyte activation and autoimmunity by the molecular adaptor Cbl-b. Nature.

[5] Chiang Y.J., Kole H.K., Brown K., Naramura M., Fukuhara S., Hu R.-J., Jang I.K., Gutkind J.S., Shevach E., Gu H. (2000). Cbl-b regulates the CD28 dependence of T-cell activation. Nature.

[6] Chiang J.Y., Jang I.K., Hodes R., Gu H. (2007). Ablation of Cbl-b provides protection against transplanted and spontaneous tumors. J. Clin. Investig..

[7] Liyasova M.S., Ma K., Lipkowitz S. (2015). Molecular pathways: Cbl proteins in tumorigenesis and antitumor immunity-opportunities for cancer treatment. Clin. Cancer Res..

[8] Wilson B.A.P., Voeller D., Smith E.A., Wamiru A., Goncharova E.I., Liu G., Lipkowitz S., O’Keefe B.R. (2021). In vitro ubiquitination platform identifies methyl ellipticiniums as ubiquitin ligase inhibitors. SLAS Discov..

[9] Jahn T., Konig G.M., Wright A.D., Worheide G., Reitner J. (1997). Manzacidin D: An unprecedented secondary metabolite from the “living fossil” sponge *Astrosclera willeyana*. Tetrahedron Lett..

[10] Williams D.H., Faulkner D.J. (1996). *N*-methylated ageliferins from the sponge *Astrosclera willeyana* from Pohnpei. Tetrahedron.

[11] Hashimoto T., Maruoka K. (2008). Synthesis of manzacidins: A stage for the demonstration of synthetic methodologies. Org. Biomol. Chem..

[12] Ma Z., Wang X., Wang X., Rodriguez R.A., Moore C.E., Gao S., Tan X., Ma Y., Rheingold A.L., Baran P.S. (2014). Asymmetric synthesis of sceptrin and massadine and evidence for biosynthetic enantiodivergence. Science.

[13] Ohfune Y., Oe K., Namba K., Shinada T. (2012). Total synthesis of manzacidins. An overview and perspective. Heterocycles.

[14] Wang X., Ma Z., Lu J., Tan X., Chen C. (2011). Asymmetric synthesis of ageliferin. J. Am. Chem. Soc..

[15] Wang X., Wang X., Tan X., Lu J., Cormier K.W., Ma Z., Chen C. (2013). Correction to a biomimetic route for construction of the [4 + 2] and [3 + 2] core skeletons of dimeric pyrrole-imidazole alkaloids and asymmetric synthesis of ageliferins. J. Am. Chem. Soc..

[16] Eder C., Proksch P., Wray V., van Soest R.W.M., Ferdinandus E., Pattisina L.A., Sudarsono S. (1999). New bromopyrrole alkaloids from the Indopacific sponge *Agelas nakamurai*. J. Nat. Prod..

[17] Hamed A.N.E., Schmitz R., Bergermann A., Totzke F., Kubbutat M., Mueller W.E.G., Youssef D.T.A., Bishr M.M., Kamel M.S., Edrada-Ebel R. (2018). Bioactive pyrrole alkaloids isolated from the Red Sea: Marine sponge *Stylissa carteri*. Z. Naturforsch. C J. Biosci..

[18] Chu M.-J., Tang X.-L., Qin G.-F., Sun Y.-T., Li L., de Voogd N.J., Li P.-L., Li G.-Q. (2017). Pyrrole derivatives and diterpene alkaloids from the South China Sea sponge *Agelas nakamurai*. Chem. Biodivers..

[19] Hattori T., Adachi K., Shizuri Y. (1997). New agelasine compound from the marine sponge *Agelas mauritiana* as an antifouling substance against macroalgae. J. Nat. Prod..

[20] Nakamura H., Wu H., Ohizumi Y., Hirata Y. (1984). Agelasine-A, -B, -C and -D, novel bicyclic diterpenoids with a 9-methladeninium unit possessing inhibitory effects on Na,K-ATPase from the Okinawa sea sponge *Agelas* sp.. Tetrahedron Lett..

[21] Marcos I.S., Garcia N., Sexmero M.J., Basabe P., Diez D., Urones J.G. (2005). Synthesis of (+)-agelasine C. A structural revision. Tetrahedron.

[22] Pettit G.R., Tang Y., Zhang Q., Bourne G.T., Arm C.A., Leet J.E., Knight J.C., Pettit R.K., Chapuis J.-C., Doubek D.L. (2013). Isolation and structures of axistatins 1–3 from the Republic of Palau marine sponge *Agelas axifera* Hentschel. J. Nat. Prod..

[23] Capon R.J., Faulkner D.J. (1984). Antimicrobial metabolites from a Pacific sponge, *Agelas* sp.. J. Am. Chem. Soc..

[24] Du K., De Mieri M., Neuburger M., Zietsman P.C., Marston A., van Vuuren S.F., Ferreira D., Hamburger M., van der Westhuizen J.H. (2015). Labdane and clerodane diterpenoids from *Colophospermum mopane*. J. Nat. Prod..

[25] Pelot K.A., Hagelthorn D.M., Hong Y.J., Tantillo D.J., Zerbe P. (2019). Diterpene synthase-catalyzed biosynthesis of distinct clerodane stereoisomers. ChemBioChem.

[26] McCloud T.G. (2010). High throughput extraction of plant, marine and fungal specimens for preservation of biologically active molecules. Molecules.

[27] Ettenberg S.A., Magnifico A., Cuello M., Nau M.M., Rubinstein Y.R., Yarden Y., Weissman A.M., Lipkowitz S. (2001). Cbl-b dependent coordinated degradation of the epidermal growth factor receptor signaling complex. J. Biol. Chem..

[28] Lorick K.L., Jensen J.P., Fang S., Ong A.M., Hatakeyama S., Weissman A.M. (1999). RING fingers mediate ubiquitin-conjugating enzyme (E2)-dependent ubiquitination. Proc. Natl. Acad. Sci. USA.

[29] Davies G.C., Ettenberg S.A., Coats A.O., Mussante M., Ravichandran S., Collins J., Nau M.M., Lipkowitz S. (2004). Cbl-b interacts with ubiquitinated proteins; differential functions of the UBA domains of c-Cbl and Cbl-b. Oncogene.

[30] Calcul L., Tenney K., Ratnam J., McKerrow J.H., Crews P. (2010). Structural variations to the 9-*N*-methyladeninium diterpenoid hybrid commonly isolated from *Agelas* sponges. Aust. J. Chem..

[31] Gordaliza M. (2009). Terpenyl-purines from the sea. Mar. Drugs.

[32] Kubota T., Iwai T., Takahashi-Nakaguchi A., Fromont J., Gonoi T., Kobayashi J. (2012). Agelasines O-U, new diterpene alkaloids with a 9-*N*-methyladenine unit from a marine sponge *Agelas* sp.. Tetrahedron.

[33] Stout E.P., Yu L.C., Molinski T.F. (2012). Antifungal diterpene alkaloids from the Caribbean sponge *Agelas citrina*: Unified configurational assignments of agelasidines and agelasines. Eur. J. Org. Chem..

[34] Yang F., Hamann M.T., Zou Y., Zhang M.-Y., Gong X.-B., Xiao J.-R., Chen W.-S., Lin H.-W. (2012). Antimicrobial metabolites from the Paracel Islands sponge *Agelas mauritiana*. J. Nat. Prod..

[35] Fathi-Afshar R., Allen T.M. (1988). Biologically active metabolites from *Agelas mauritiana*. Can. J. Chem..

[36] Ohba M., Iizuka K., Ishibashi H., Fujii T. (1997). Synthesis and absolute configurations of the marine sponge purines (+)-agelasimine-A and (+)-agelasimine-B. Tetrahedron.

