# Supplementary material for: Agelasine Diterpenoids and Cbl-b Inhibitory Ageliferins from the Coralline Demosponge Astrosclera willeyana
Source: Mar Drugs. 2021 Jun 24;19(7):361. doi: 10.3390/md19070361 (PMC8307156; doi:10.3390/md19070361)
Supplement: Supplementary file 1 [file marinedrugs-19-00361-s001.zip › marinedrugs-1271726-supplementary.pdf]

***Supporting Information***

# Agelasine Diterpenoids and Cbl-b Inhibitory Ageliferins from the Coralline Demosponge *Astrosclera willeyana*

Wei Jiang,[†,‡] Dongdong Wang,[‡] Brice A. P. Wilson,[‡] Unwoo Kang,[‡] Heidi R. Bokesch,[‡,§] Emily A. Smith,[‡,§] Antony Wamiru, [‡,§] Katya Goncharova, [‡,○] Donna Voeller,[⊥] Stanley Lipkowitz,[⊥] Barry R. O'Keefe,[‡,|] Kirk R. Gustafson*[, ‡]

[†]Marine Science & Technology Institute, College of Environmental Science & Engineering, Yangzhou University, Yangzhou, Jiangsu 225127, People's Republic of China

[‡]Molecular Targets Program, Center for Cancer Research, National Cancer Institute, Frederick, Maryland 21702-1201, United States

[§]Basic Science Program, Leidos Biomedical Research, Inc., Frederick National Laboratory for Cancer Research, Frederick, Maryland 21702-1201, United States

[⊥]Women's Malignancies Branch, Center for Cancer Research, National Cancer Institute, Bethesda, Maryland 20892, United States

[○]Advanced Biomedical Computational Science, Frederick National Laboratory for Cancer Research, Frederick, Maryland 21702-1201, United States

[|]Natural Products Branch, Developmental Therapeutics Program, Division of Cancer Treatment and Diagnosis, National Cancer Institute, Frederick, Maryland 21701-1201, United States

**Supporting Information**


**Figure S1.** $^{1}H$ NMR spectrum (600 MHz) of Agelasine W (**1**) in $CD_3OD$.

**Figure S2.** $^{13}C$ NMR spectrum (150 MHz) of Agelasine W (**1**) in $CD_3OD$.

**Figure S3.** HSQC spectrum of Agelasine W (**1**) in $CD_3OD$.

**Figure S4.** HMBC spectrum of Agelasine W (**1**) in $CD_3OD$.

**Figure S5.** COSY spectrum of Agelasine W (**1**) in $CD_3OD$.

**Figure S6.** NOESY spectrum of Agelasine W (**1**) in $CD_3OD$.

**Figure S7.** HRESIMS of Agelasine W (**1**).

**Figure S8.** IR spectrum of Agelasine W (**1**).

**Figure S9.** UV spectrum of Agelasine W (**1**).

**Figure S10.** $^{1}H$ NMR spectrum (600 MHz) of Agelasine X (**2**) in $CD_3OD$.

**Figure S11.** $^{13}C$ NMR spectrum (150 MHz) of Agelasine X (**2**) in $CD_3OD$.

**Figure S12.** HSQC spectrum of Agelasine X (**2**) in $CD_3OD$.

**Figure S13.** HMBC spectrum of Agelasine X (**2**) in $CD_3OD$.

**Figure S14.** COSY spectrum of Agelasine X (**2**) in $CD_3OD$.

**Figure S15.** HRESIMS of Agelasine X (**2**).

**Figure S16.** IR spectrum of Agelasine X (**2**).

**Figure S17.** UV spectrum of Agelasine X (**2**).

**Figure S18.** $^{1}H$ NMR spectrum (600 MHz) of Agelasine Y (**3**) in $CD_3OD$.

**Figure S19.** $^{13}C$ NMR spectrum (150 MHz) of Agelasine Y (**3**) in $CD_3OD$.

**Figure S20.** HSQC spectrum of Agelasine Y (**3**) in $CD_3OD$.

**Figure S21.** HMBC spectrum of Agelasine Y (**3**) in $CD_3OD$.

**Figure S22.** COSY spectrum of Agelasine Y (**3**) in $CD_3OD$.

**Figure S23.** NOESY spectrum of Agelasine Y (**3**) in $CD_3OD$.

**Figure S24.** HRESIMS of Agelasine Y (**3**).

**Figure S25.** IR spectrum of Agelasine Y (**3**).

**Figure S26.** UV spectrum of Agelasine Y (**3**).

**Figure S27.** $^{1}H$ NMR spectrum (600 MHz) of *N*(1)-methylisoageliferin (**4**) in $CD_3OD$.

**Figure S28.** $^{13}C$ NMR spectrum (150 MHz) of *N*(1)-methylisoageliferin (**4**) in $CD_3OD$.

**Figure S29.** HSQC spectrum of *N*(1)-methylisoageliferin (**4**) in $CD_3OD$.

**Figure S30.** HMBC spectrum of *N*(1)-methylisoageliferin (**4**) in $CD_3OD$.

**Figure S31.** COSY spectrum of *N*(1)-methylisoageliferin (**4**) in $CD_3OD$.

**Figure S32.** ECD spectrum of *N*(1)-methylisoageliferin (**4**).

**Figure S33.** HRESIMS of *N*(1)-methylisoageliferin (**4**).

**Figure S34.** IR spectrum of *N*(1)-methylisoageliferin (**4**).

**Figure S35.** UV spectrum of *N*(1)-methylisoageliferin (**4**).

**Table S1.** $^{1}$H NMR (600 MHz) data for compounds **5-10** in $CD_3OD$.

**Table S2.** $^{13}$C NMR (150 MHz) data for compound **5-10** in $CD_3OD$.

**Table S3.** $^{13}$C NMR (150 MHz) data for compound **3** and closely related clerodane diastereomers in $CDCl_3$.

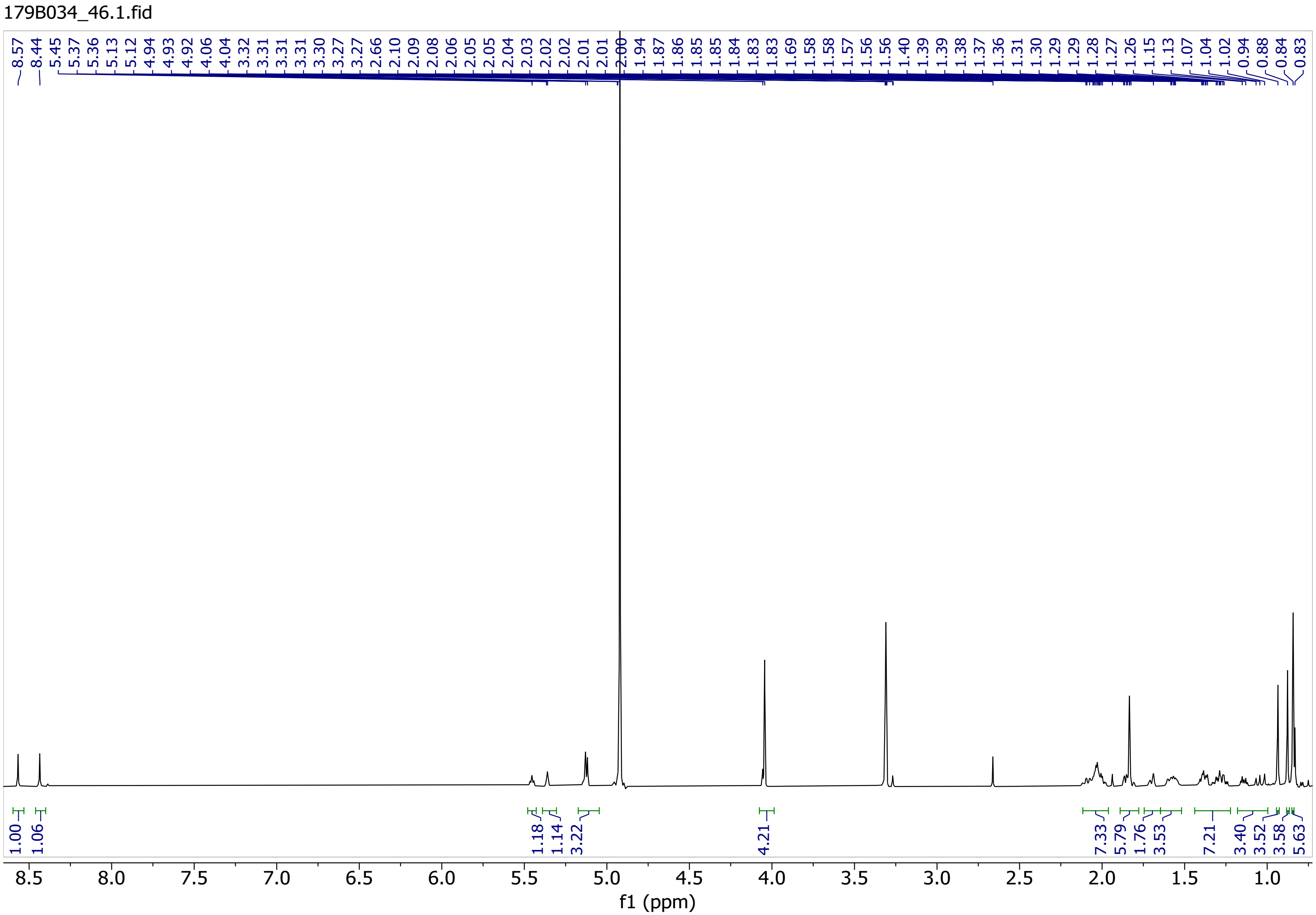


**Figure S1.** $^{1}H$ NMR spectrum (600 MHz) of Agelasine W (**1**) in $CD_3OD$.

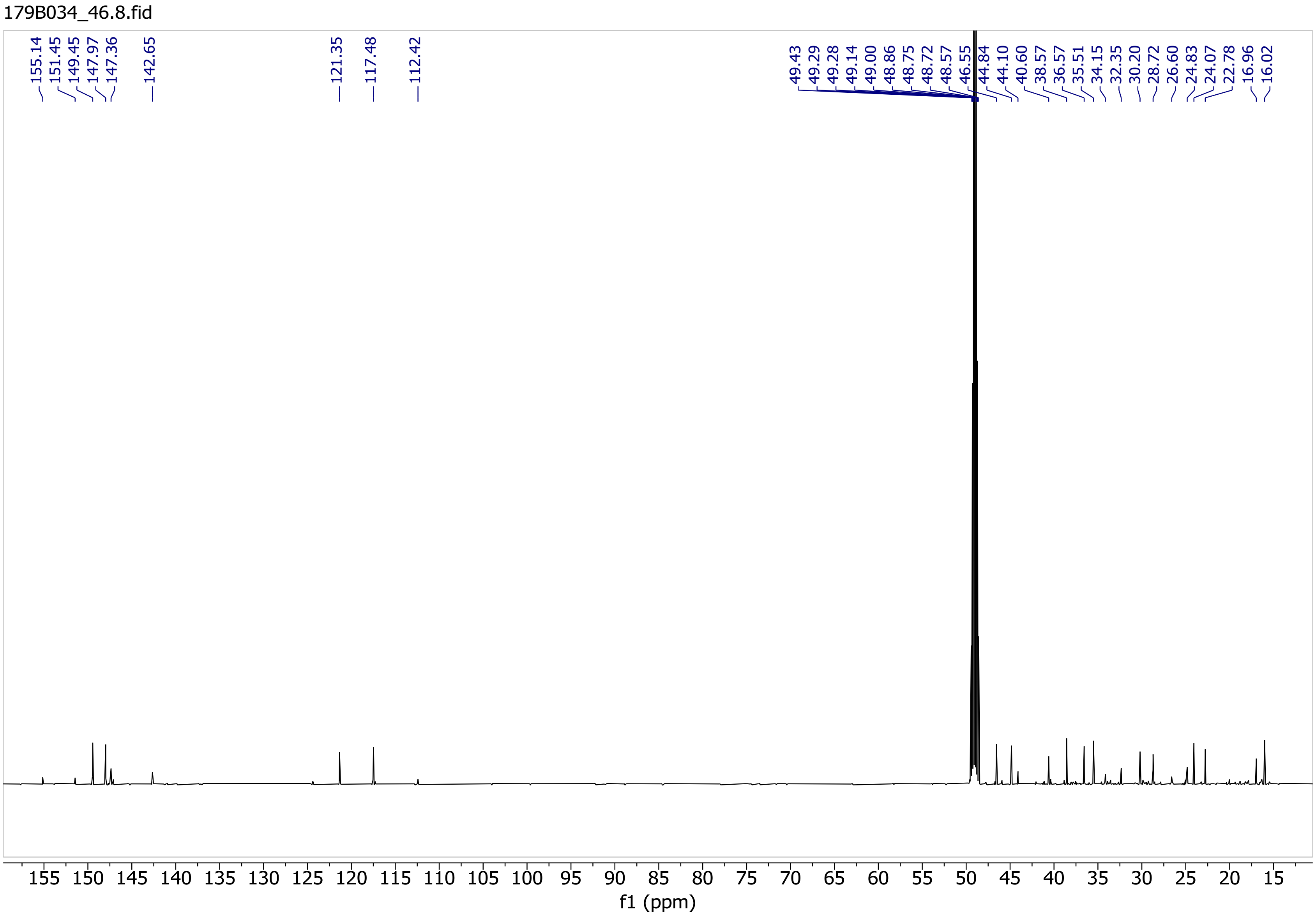


**Figure S2.** $^{13}C$ NMR spectrum (150 MHz) of Agelasine W (**1**) in $CD_3OD$.

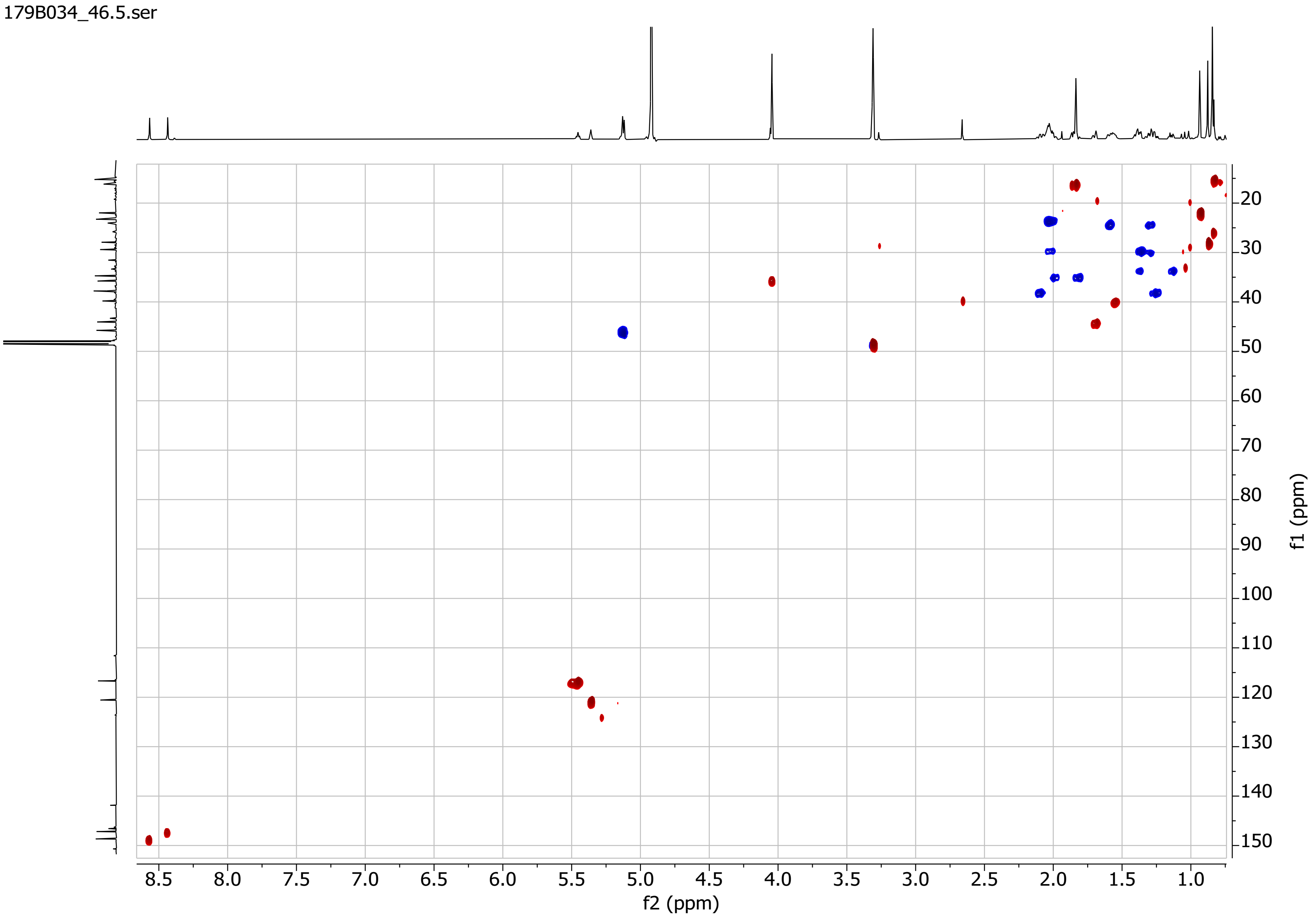


**Figure S3.** HSQC spectrum of Agelasine W (**1**) in $CD_3OD$.

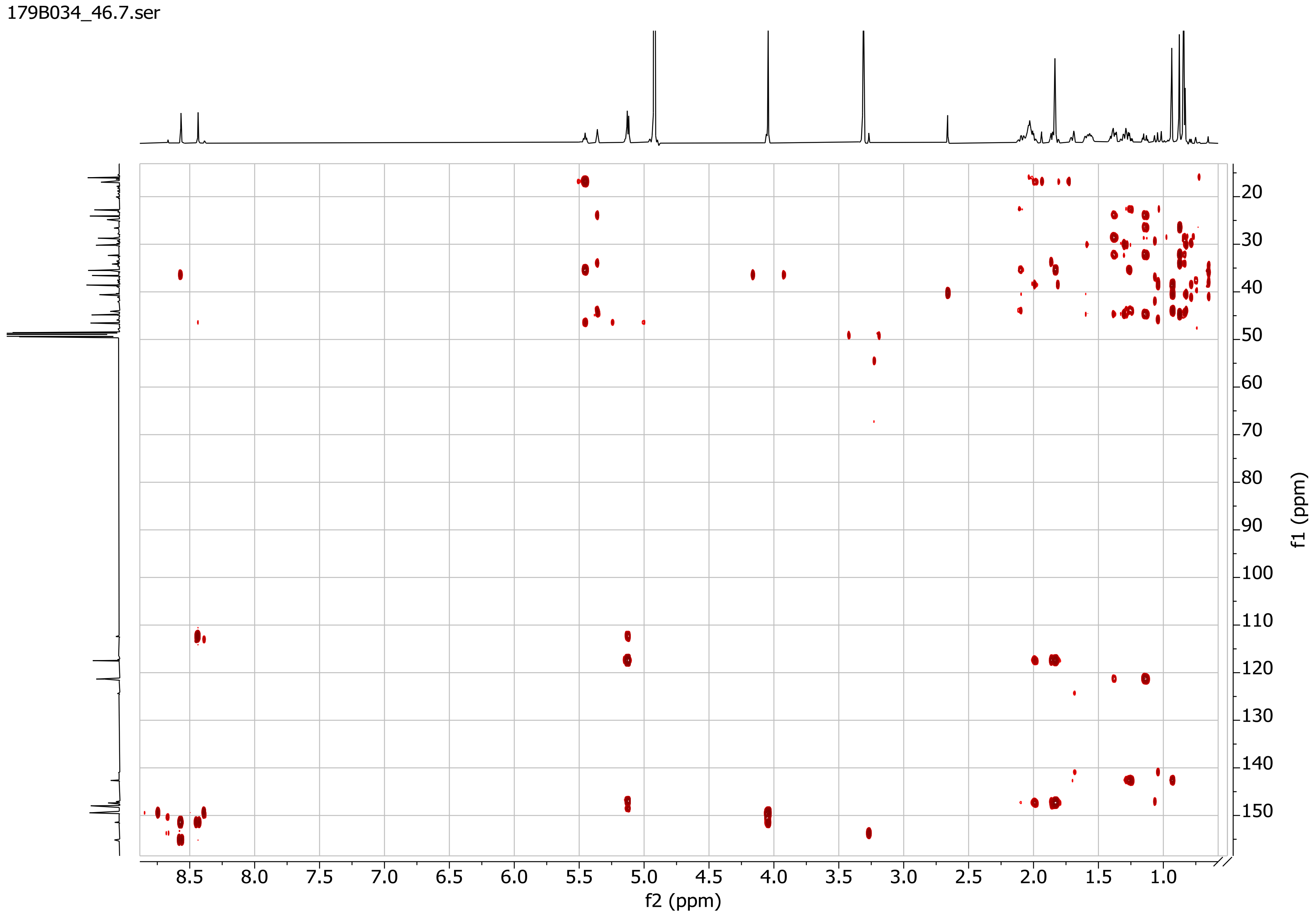


**Figure S4.** HMBC spectrum of Agelasine W (**1**) in $CD_3OD$.

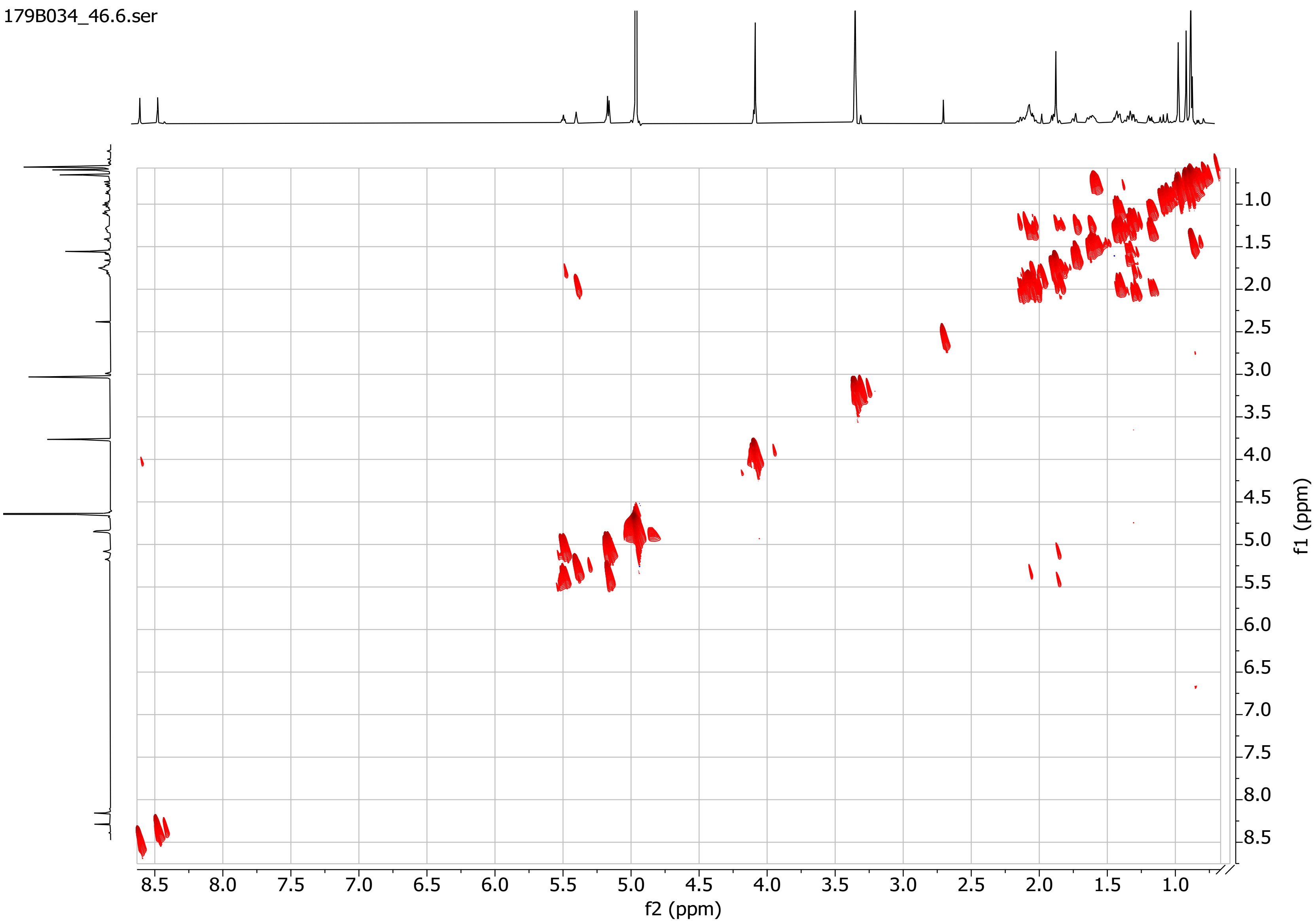


**Figure S5.** COSY spectrum of Agelasine W (**1**) in $CD_3OD$.

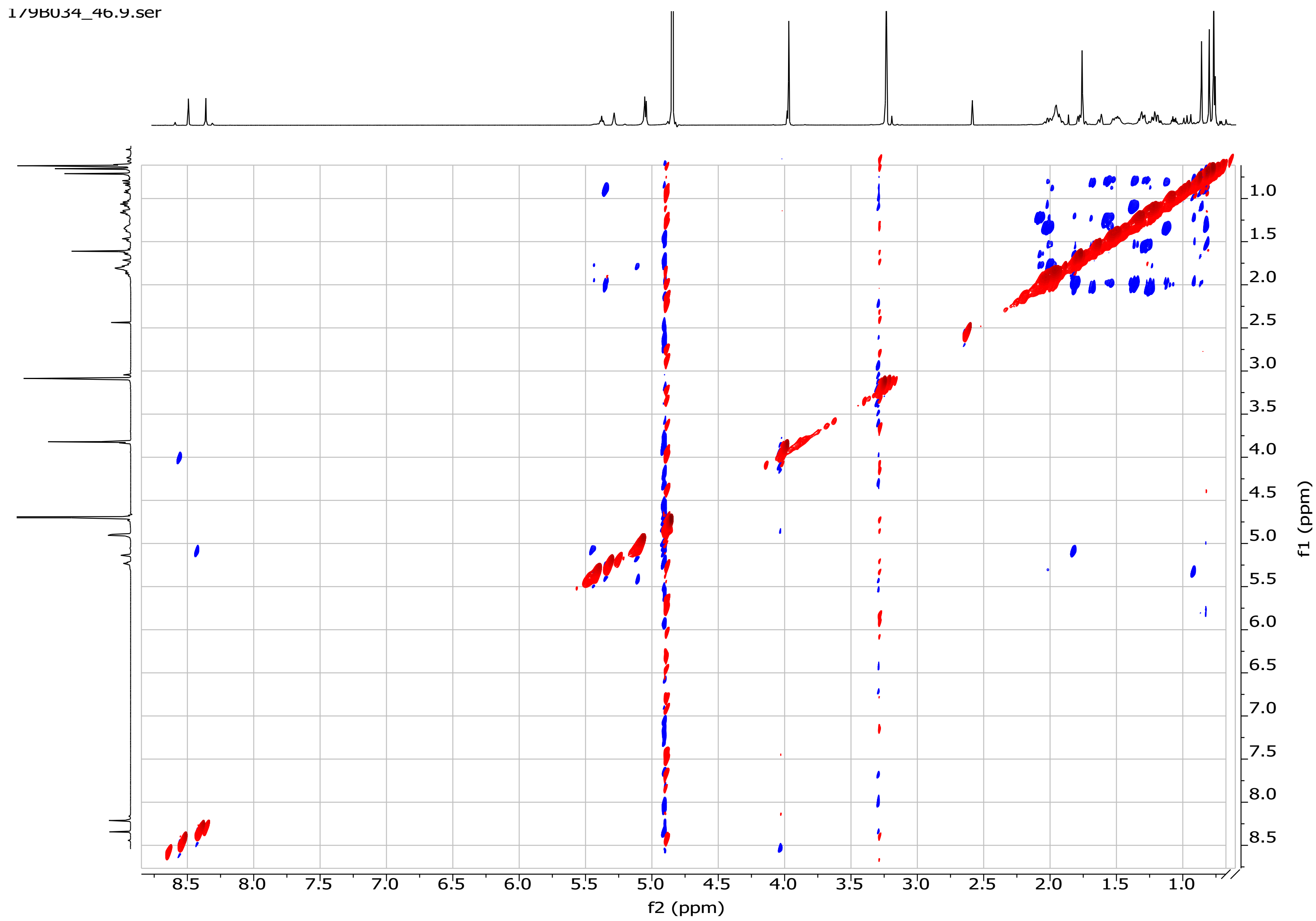


**Figure S6.** NOESY spectrum of Agelasine W (**1**) in $CD_3OD$.

# Analysis Report

Agilent Trusted Answers

## Sample Information

| | | | |
|---|---|---|---|
| **Name** | 179B034-46 | **Data File Path** | C:\Users\bokeschh\Desktop\QTOF-DATAANALYSIS-ADMIN by HEIDIB\Wei\102919\179B034-46.d |
| **Sample ID** | | **Acq. Time (Local)** | 10/29/2019 8:47:47 AM (UTC-05:00) |
| **Instrument** | Instrument 1 | **Method Path (Acq)** | D:\MassHunter\Methods\FIA_SM_LowFlow.m |
| **MS Type** | QTOF | **Version (Acq SW)** | 6200 series TOF/6500 series Q-TOF B.09.00 (B9044.1 SP1) |
| **Inj. Vol. (ul)** | 1 | **IRM Status** | Some ions missed |
| **Position** | Vial 16 | **Method Path (DA)** | |
| **Plate Pos.** | | **Target Source Path** | |
| **Operator** | | **Result Summary** | |

## Sample Chromatograms

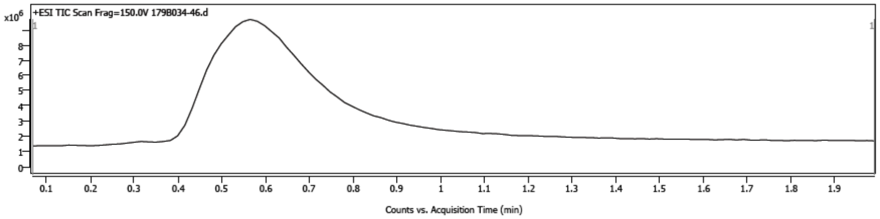


## Sample Spectra

**+ Scan (rt: 0.399-0.830 min)** **C26 H40 N5**

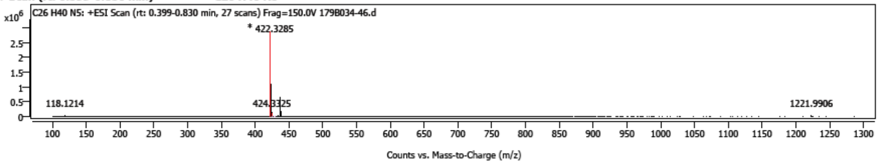


*Spectrum Identification Table*

| Best | ID Source | Name | Formula | Species | m/z | Diff (ppm) | CAS | Score | Score (Lib) | Score (DB) | Score (MFG) | Lib/DB |
|---|---|---|---|---|---|---|---|---|---|---|---|---|
| Yes | MFG | | C26 H40 N5 | M+ | 422.3285 | 0.31 | | 84.22 | | | 84.22 | |

**MassHunter Qual 10.0**
**(End of Report)**



**Figure S7.** HRESIMS spectrum of Agelasine W (**1**).

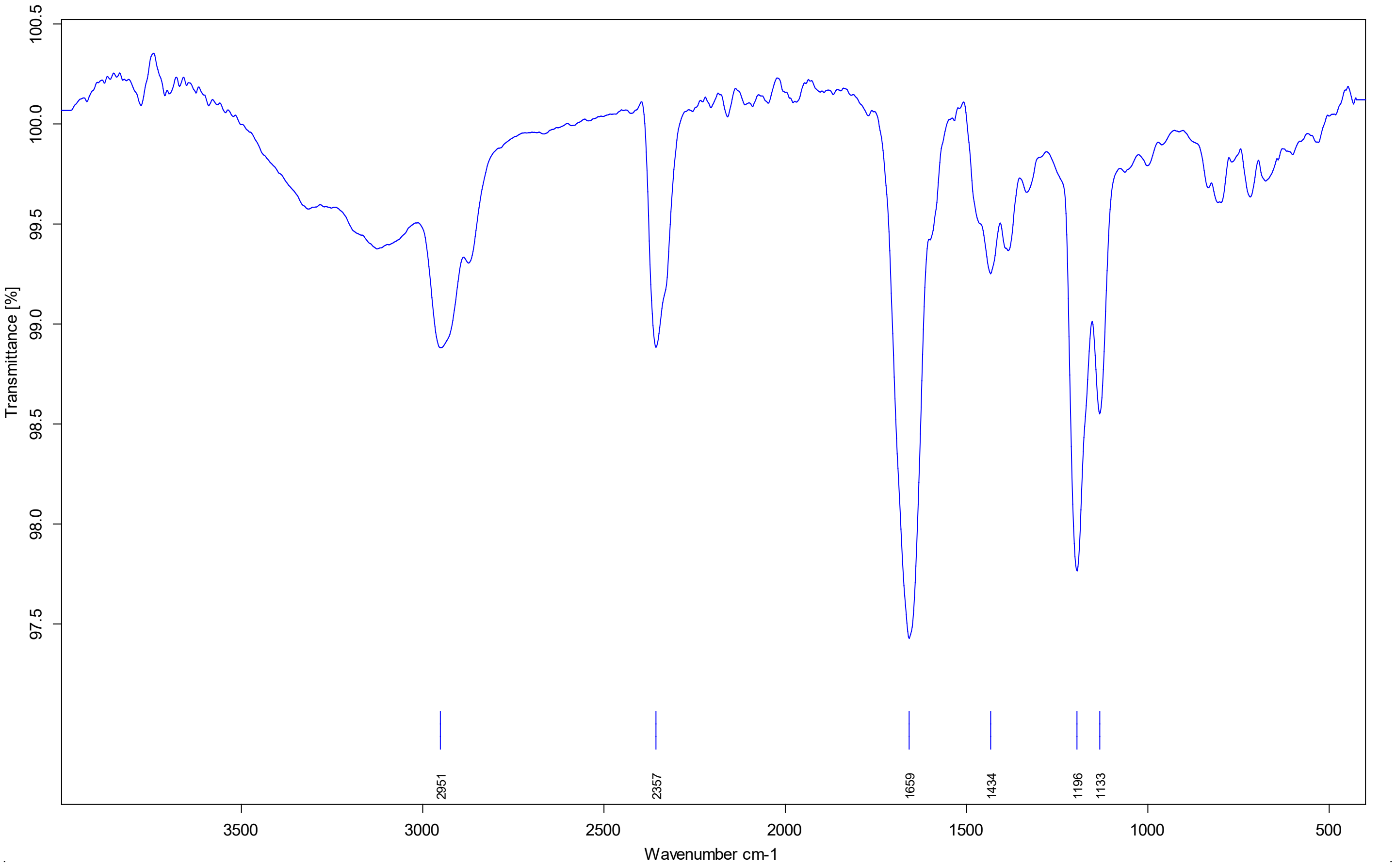


**Figure S8.** IR spectrum (neat) of Agelasine W (**1**).

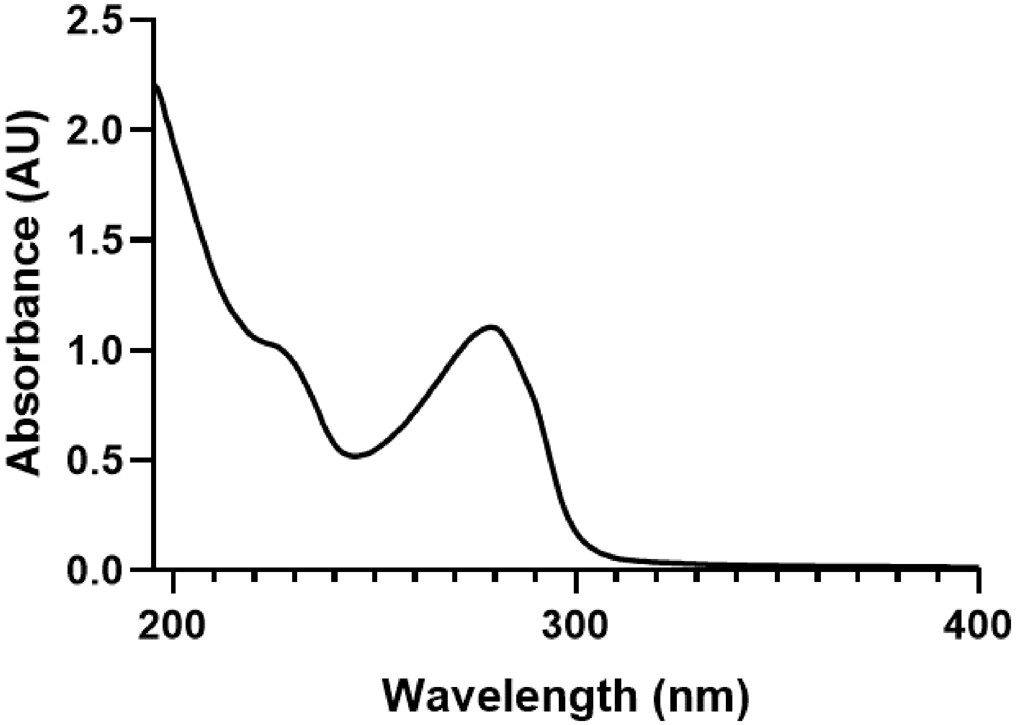


**Figure S9.** UV spectrum of Agelasine W (**1**).

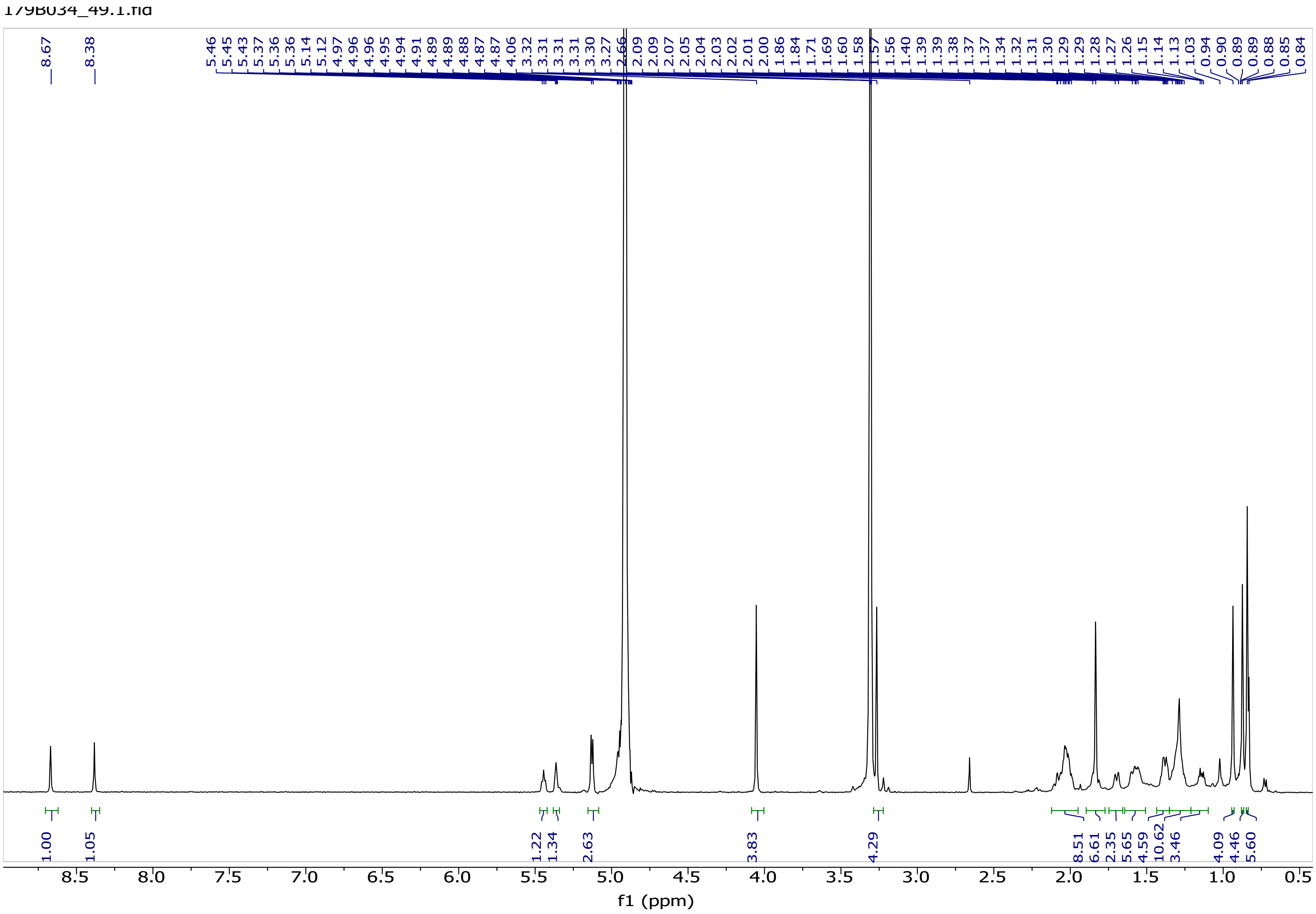


**Figure S10.** $^1H$ NMR spectrum (600 MHz) of Agelasine X (**2**) in $CD_3OD$.

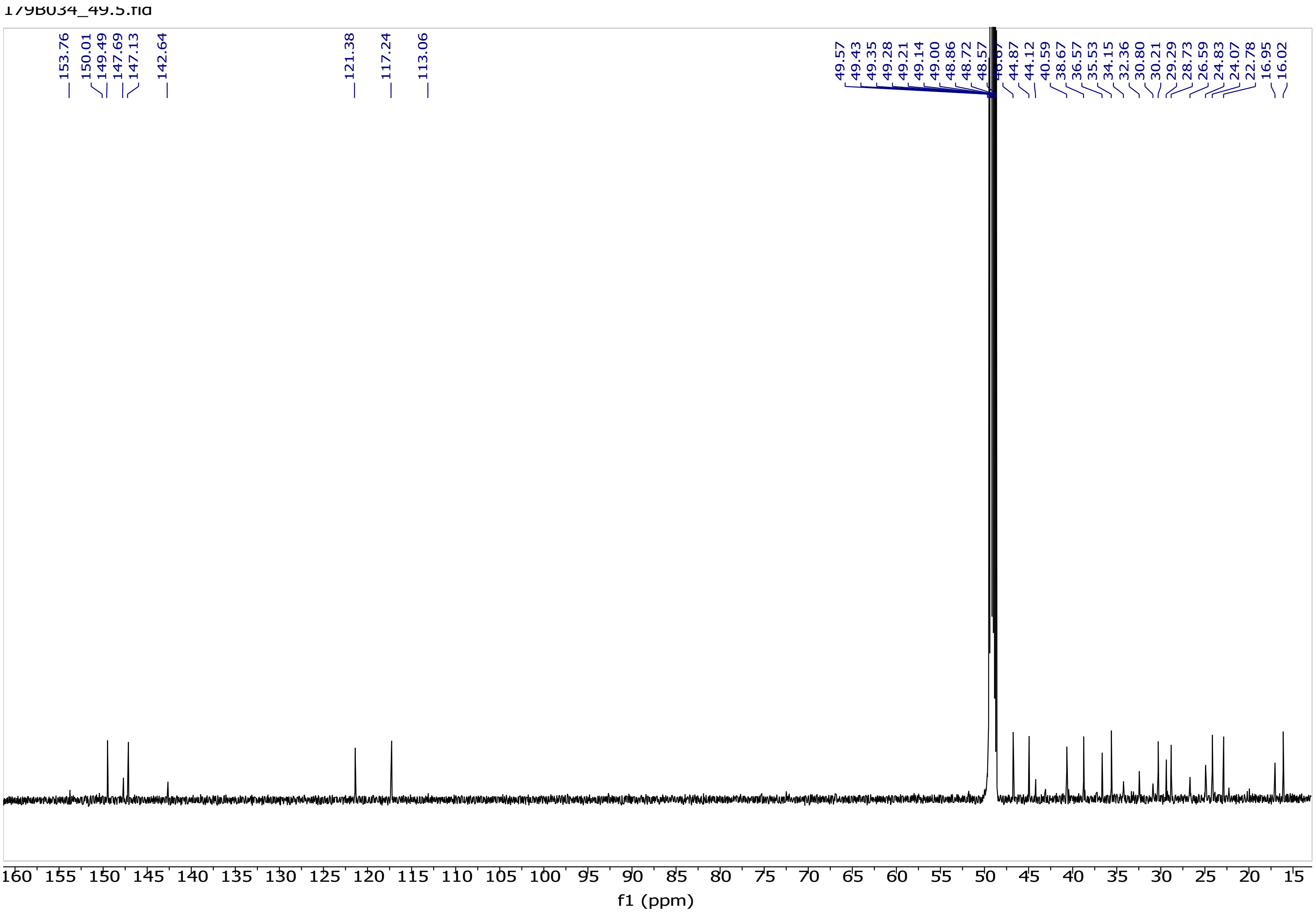


**Figure S11.** $^{13}$C NMR spectrum (150 MHz) of Agelasine X (**2**) in $CD_3OD$.

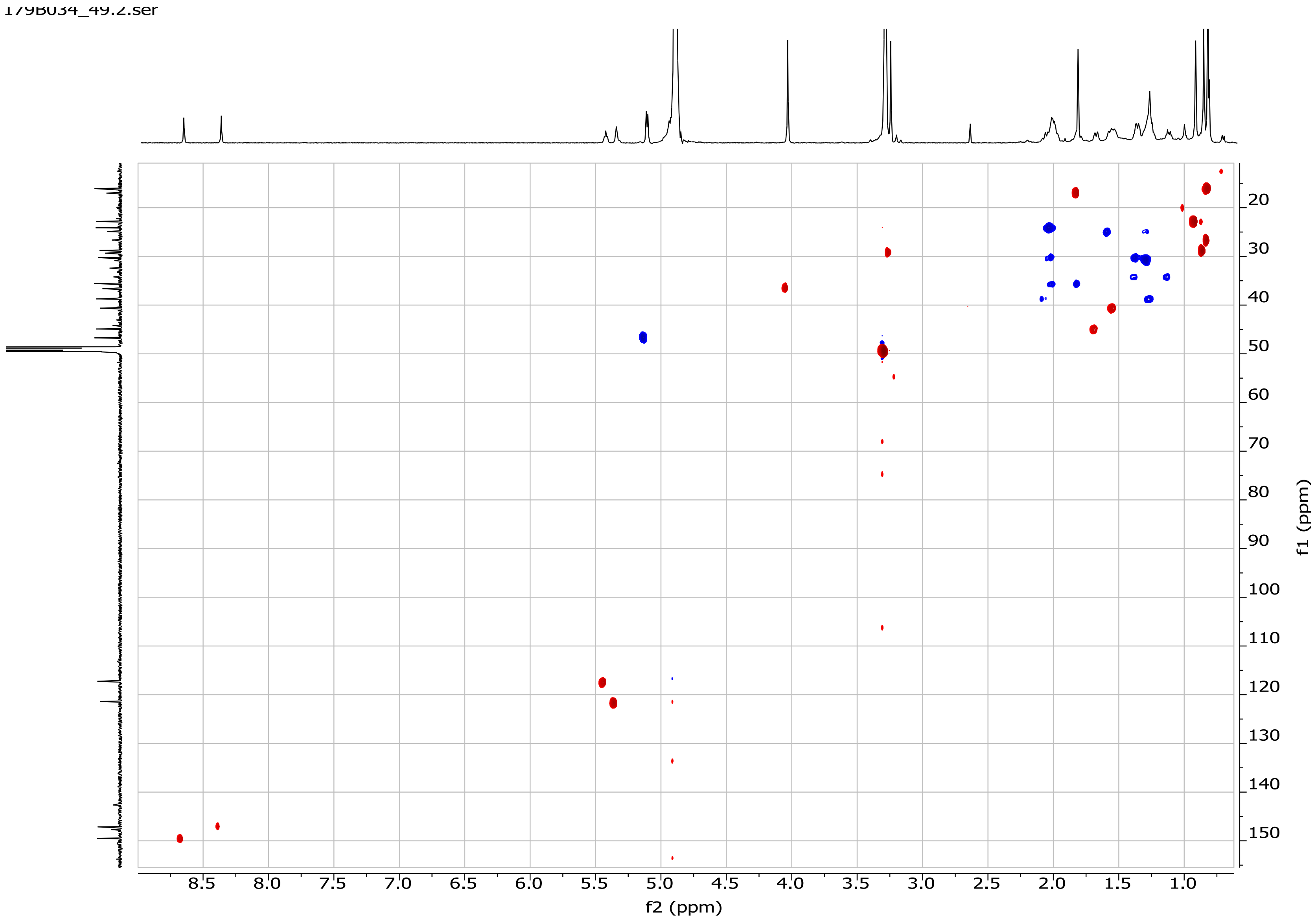


**Figure S12.** HSQC spectrum of Agelasine X (**2**) in $CD_3OD$.

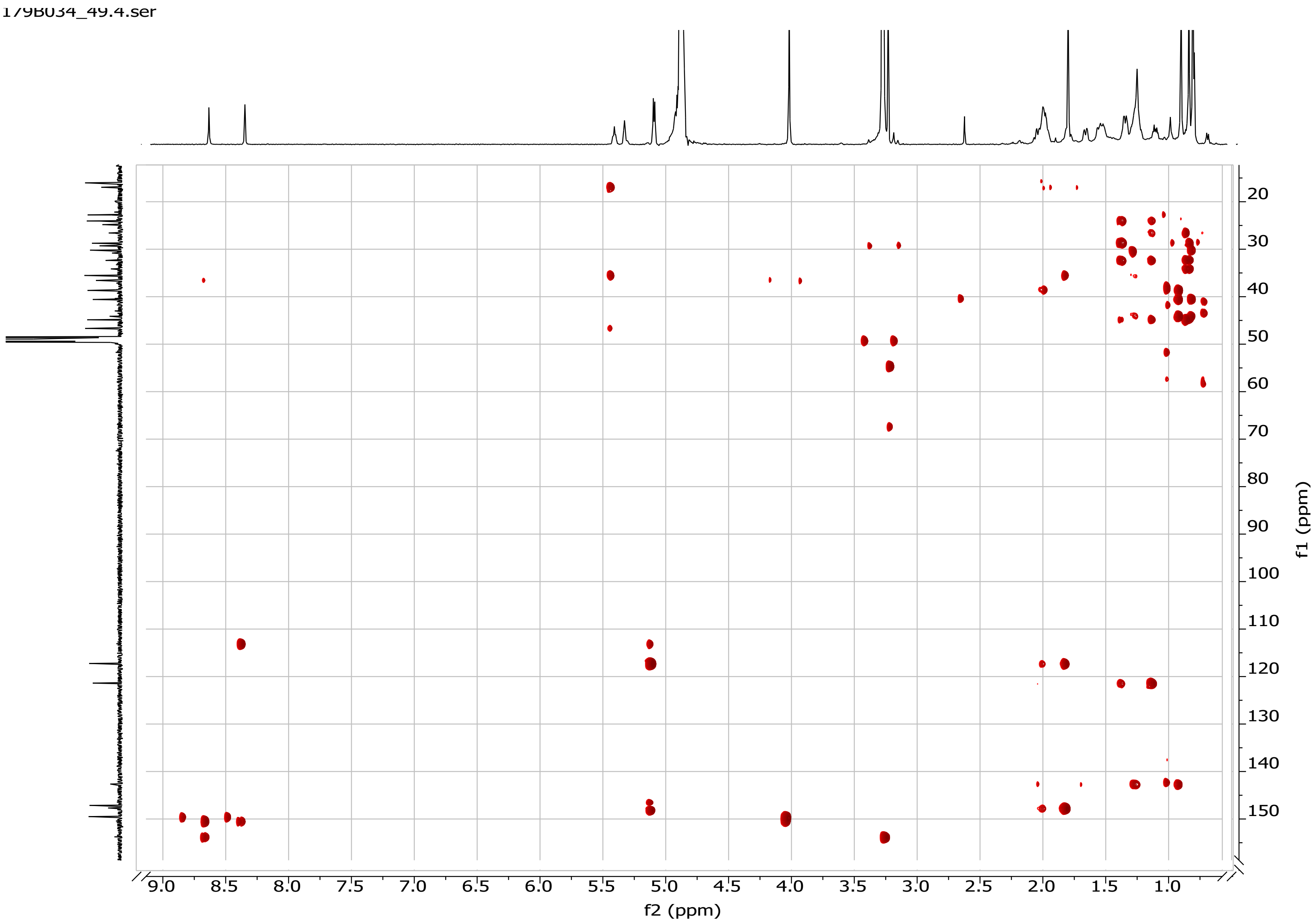


**Figure S13.** HMBC spectrum of Agelasine X (**2**) in $CD_3OD$.

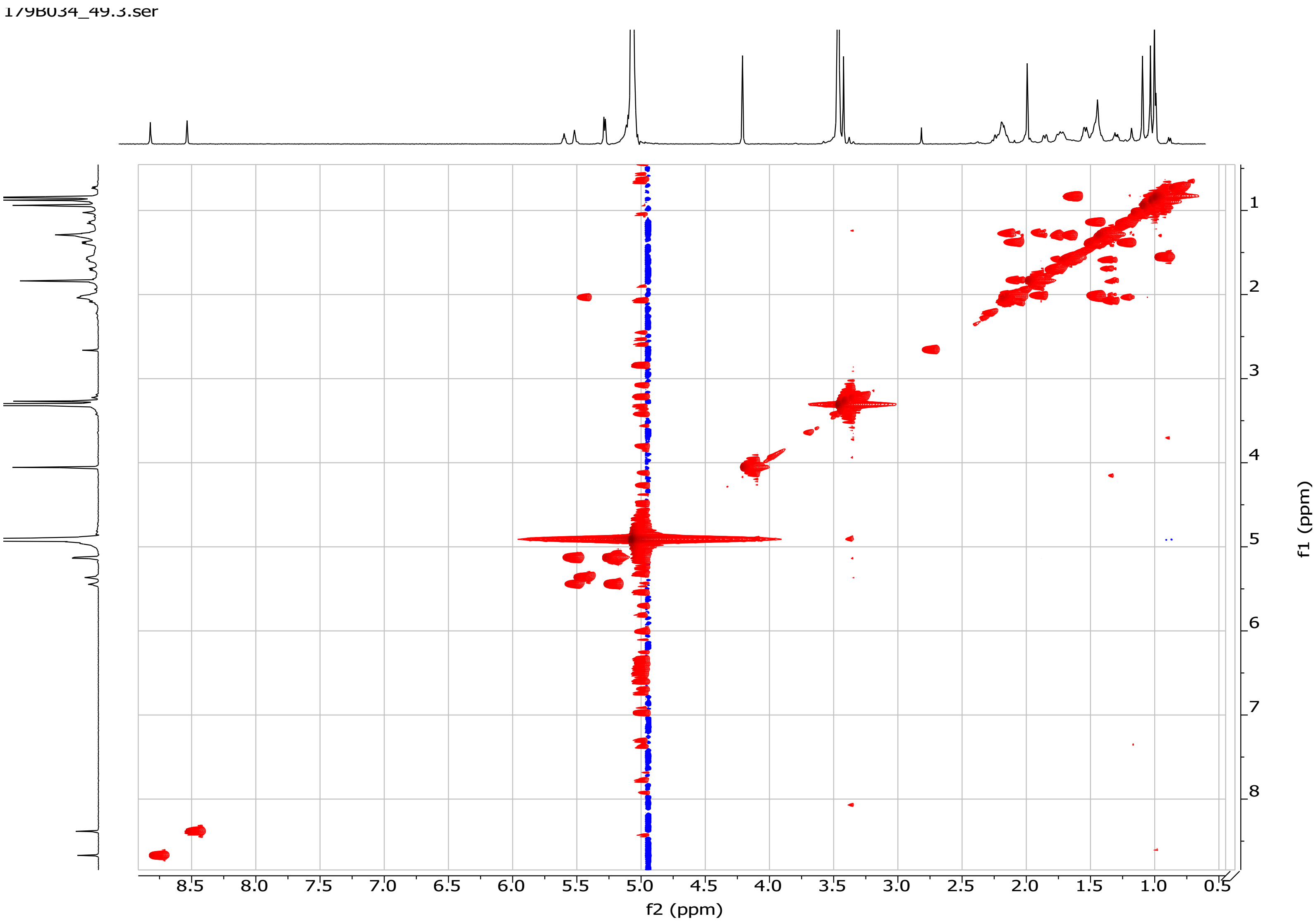


**Figure S14.** COSY spectrum of Agelasine X (**2**) in $CD_3OD$.

# Analysis Report

Agilent | Trusted Answers

## Sample Information

| | | | |
|---|---|---|---|
| **Name** | 179B034-49 | **Data File Path** | C:\Users\bokeschh\Desktop\QTOF-DATAANALYSIS-ADMIN by HEIDIB\Wei\102919\179B034-49.d |
| **Sample ID** | | **Acq. Time (Local)** | 10/29/2019 8:58:41 AM (UTC-05:00) |
| **Instrument** | Instrument 1 | **Method Path (Acq)** | D:\MassHunter\Methods\FIA_SM_LowFlow.m |
| **MS Type** | QTOF | **Version (Acq SW)** | 6200 series TOF/6500 series Q-TOF B.09.00 (B9044.1 SP1) |
| **Inj. Vol. (ul)** | 1 | **IRM Status** | Some ions missed |
| **Position** | Vial 18 | **Method Path (DA)** | C:\Users\bokeschh\Desktop\QTOF-DATAANALYSIS-ADMIN by HEIDIB\Wei\102919\179B034-49.d\Results\Qual\Version4\HB.m |
| **Plate Pos.** | | **Target Source Path** | |
| **Operator** | | **Result Summary** | |

## Sample Chromatograms

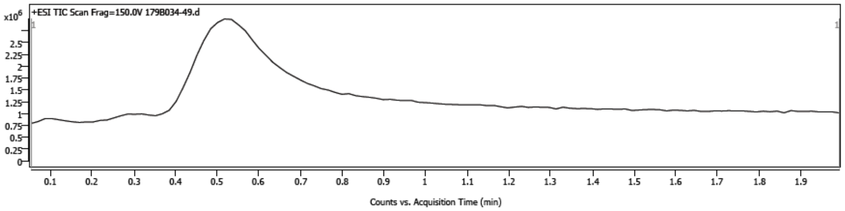


## Sample Spectra

**+ Scan (rt: 0.385-0.783 min)** **C27 H42 N5**

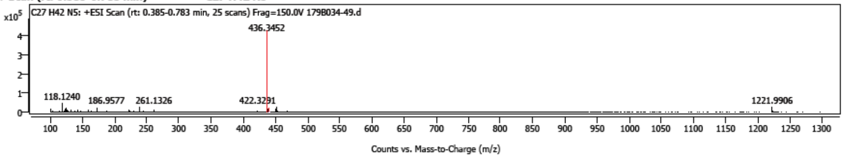


*Spectrum Identification Table*

| Best | ID Source | Name | Formula | Species | m/z | Diff (ppm) | CAS | Score | Score (Lib) | Score (DB) | Score (MFG) | Lib/DB |
|---|---|---|---|---|---|---|---|---|---|---|---|---|
| Yes | MFG | | C27 H42 N5 | M+ | 436.3452 | 3.76 | | 92.33 | | | 92.33 | |

**MassHunter Qual 10.0**
**(End of Report)**



**Figure S15.** HRESIMS spectrum of Agelasine X (**2**).

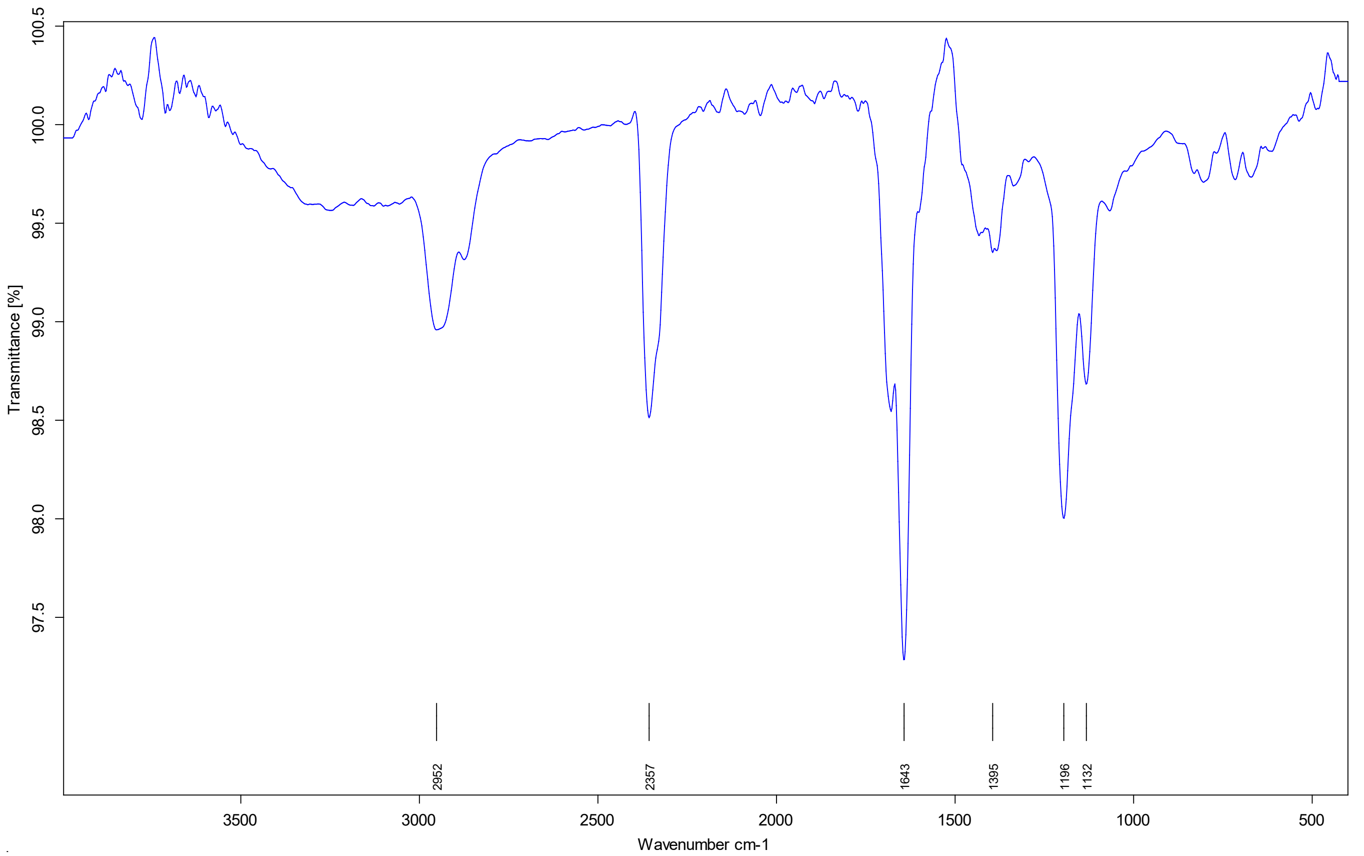


**Figure S16.** IR spectrum (neat) of Agelasine X (**2**).

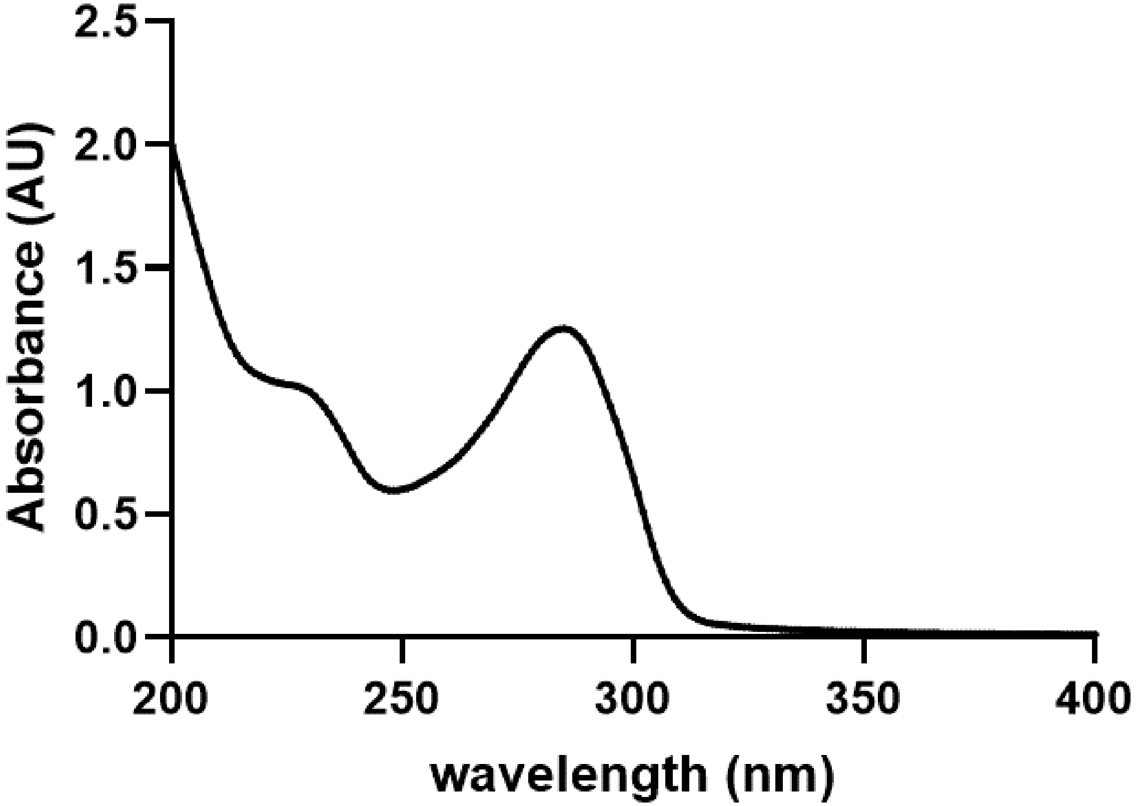


**Figure S17.** UV spectrum of Agelasine X (**2**).

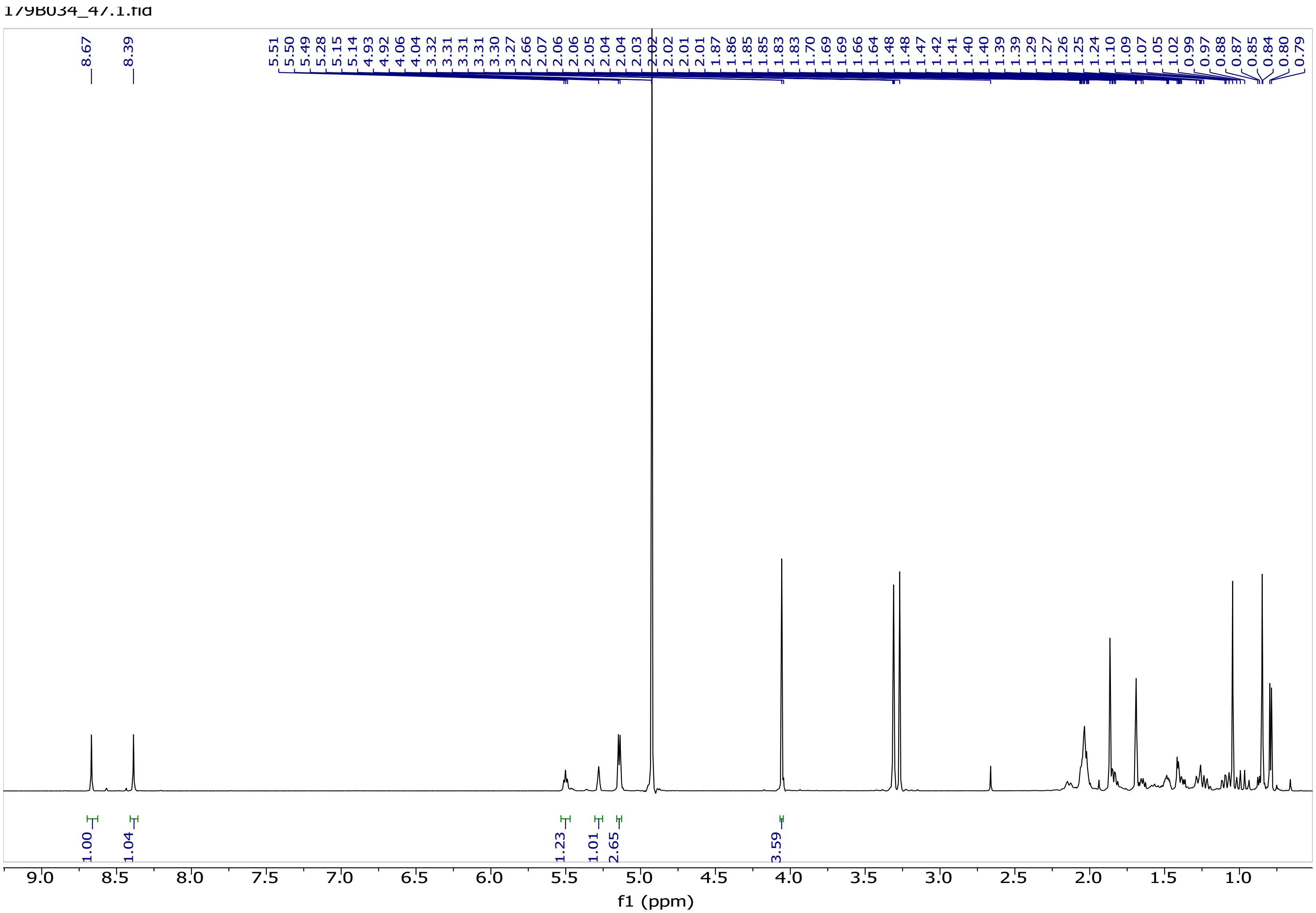


**Figure S18.** $^{1}$H NMR spectrum (600 MHz) of Agelasine Y (**3**) in $CD_3OD$.

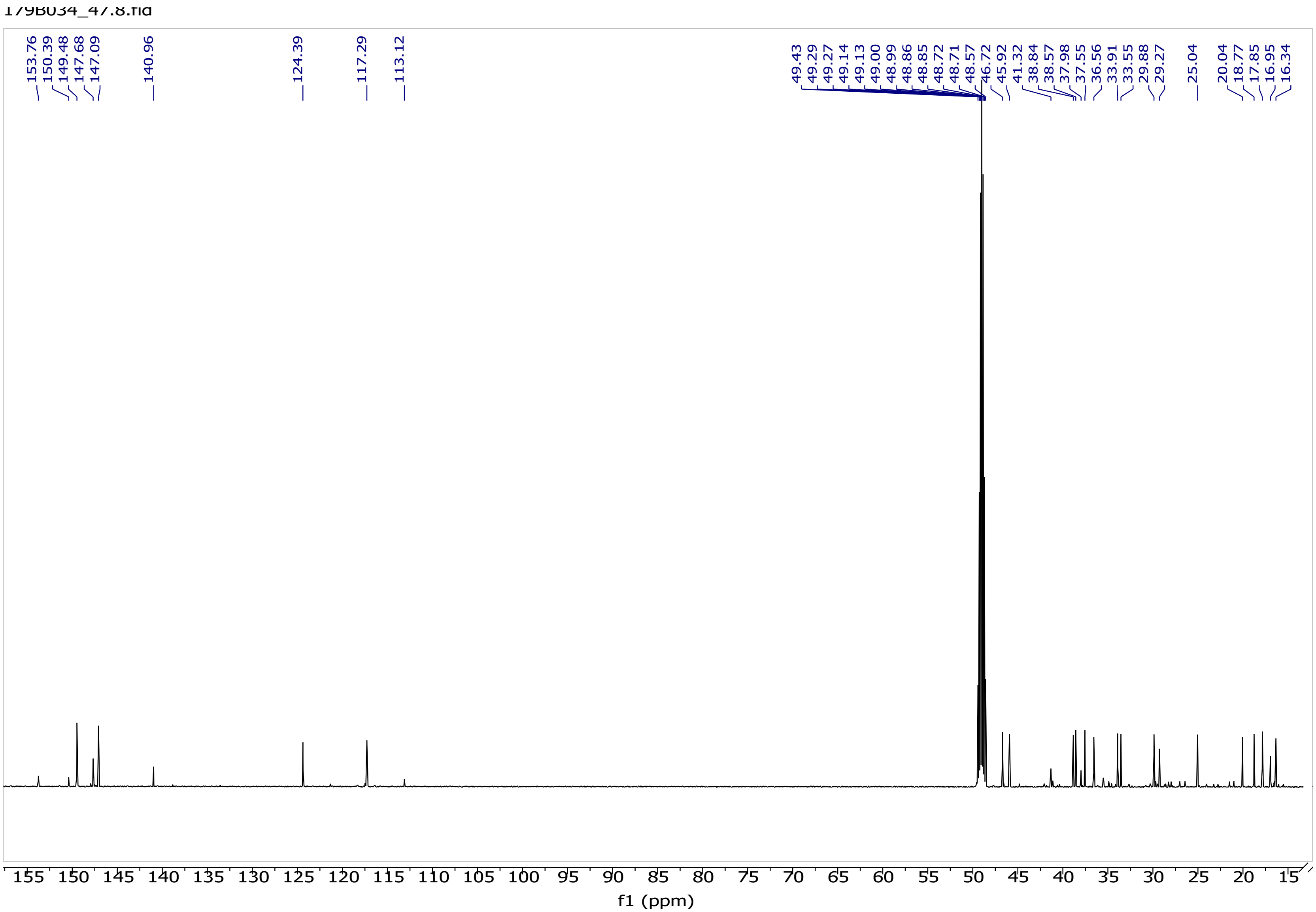


**Figure S19.** $^{13}C$ NMR spectrum (150 MHz) of Agelasine Y (**3**) in $CD_3OD$.

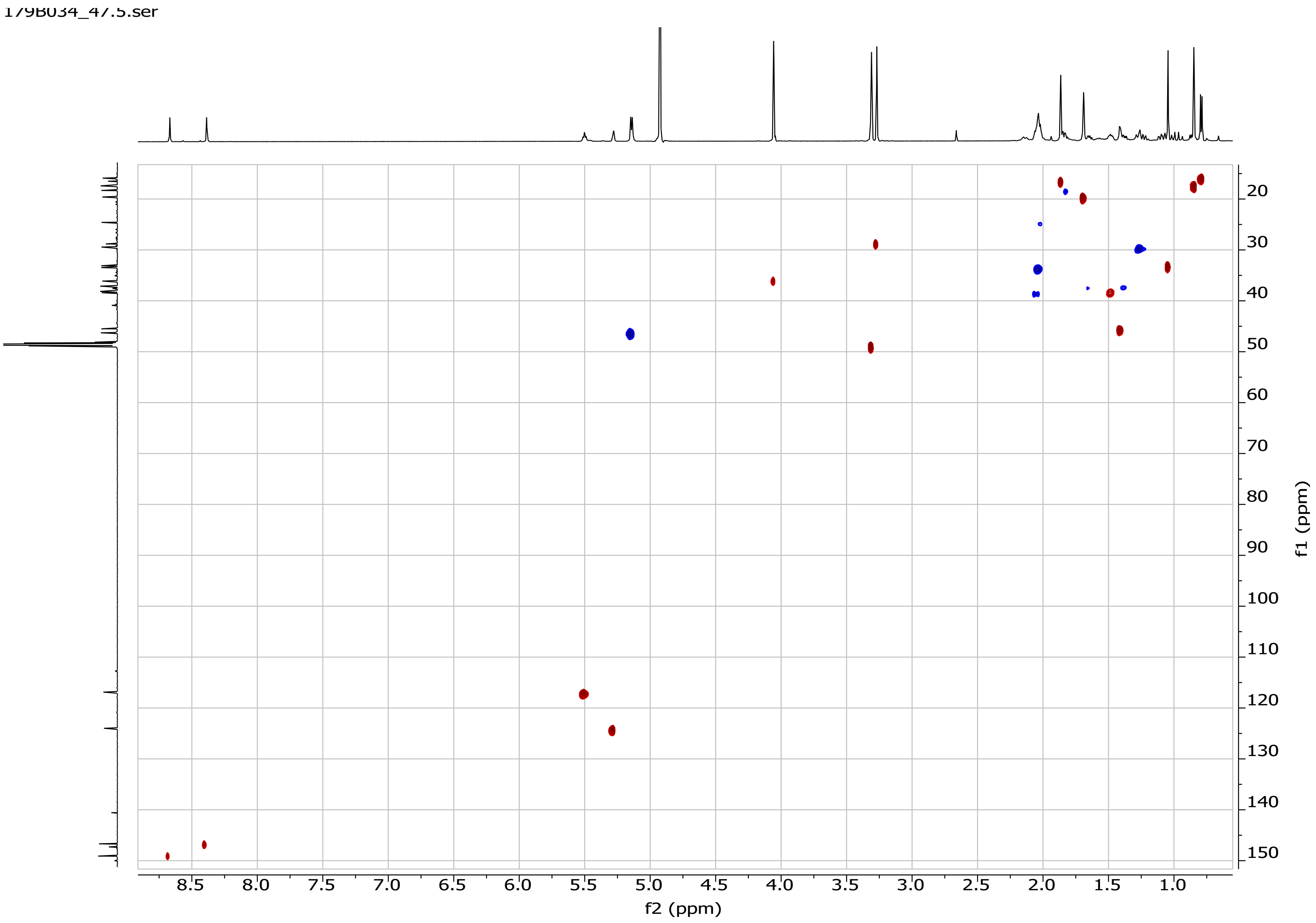


**Figure S20.** HSQC spectrum of Agelasine Y (**3**) in $CD_3OD$.

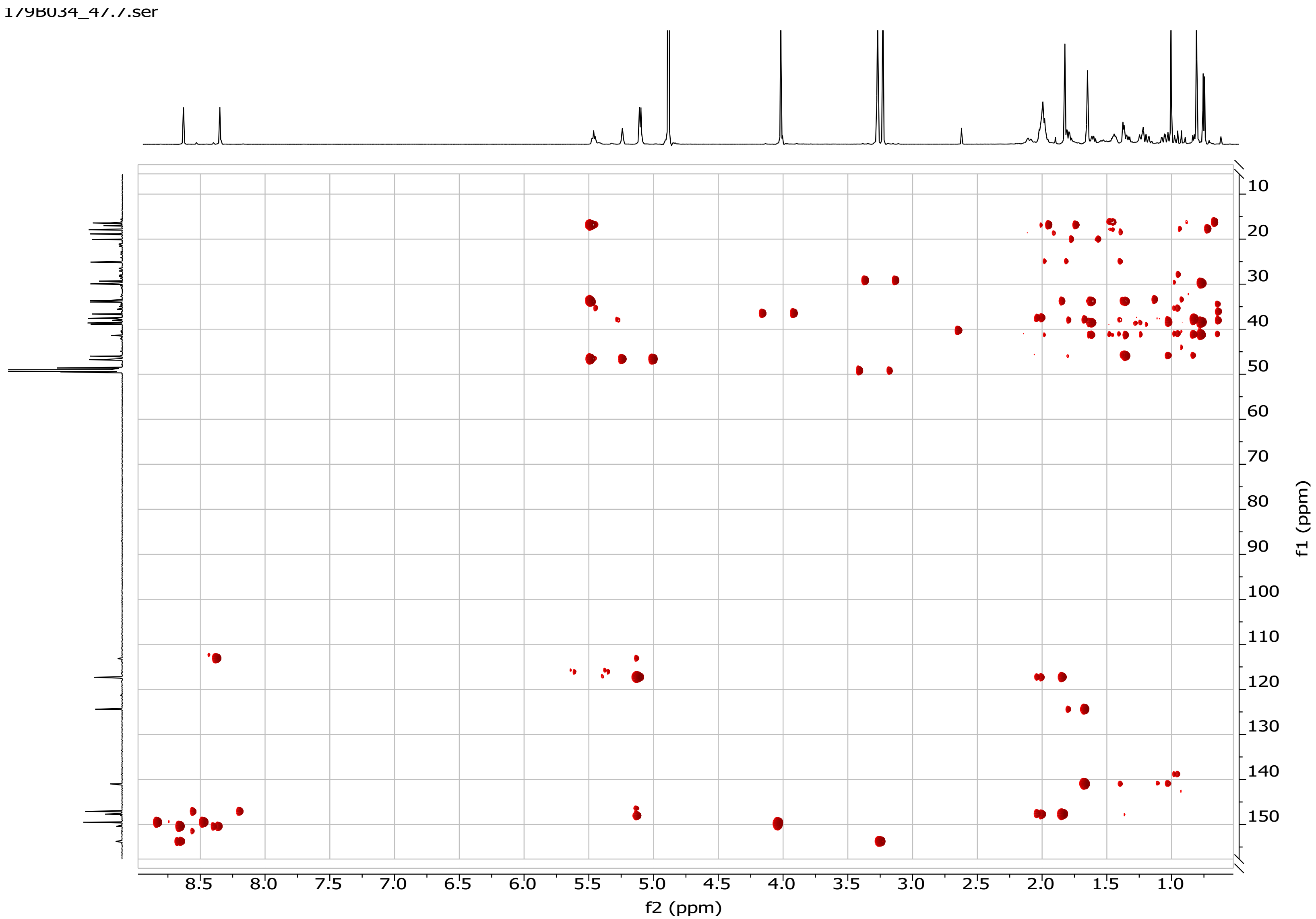


**Figure S21.** HMBC spectrum of Agelasine Y (**3**) in $CD_3OD$.

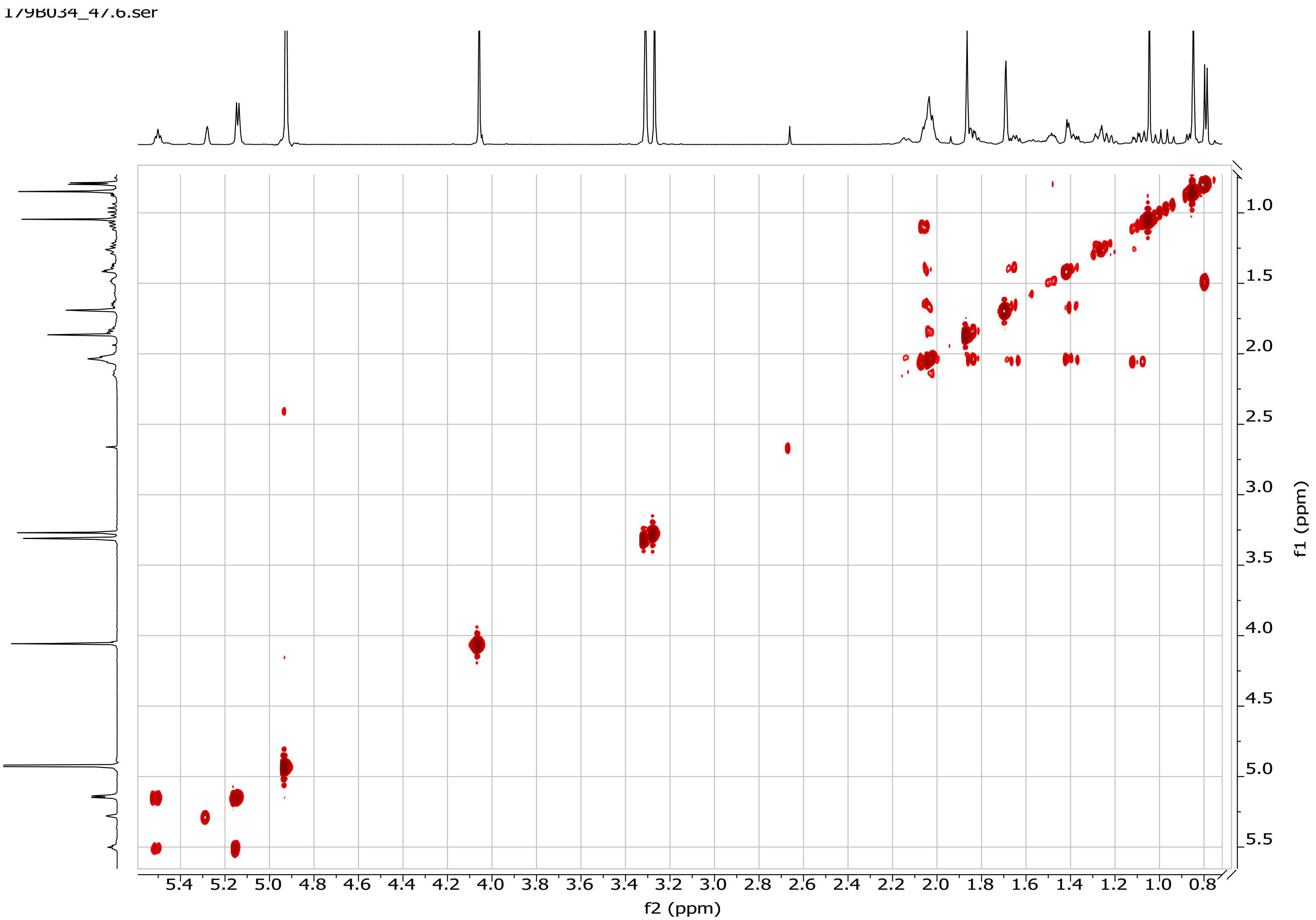


**Figure S22.** COSY spectrum of Agelasine Y (**3**) in $CD_3OD$.

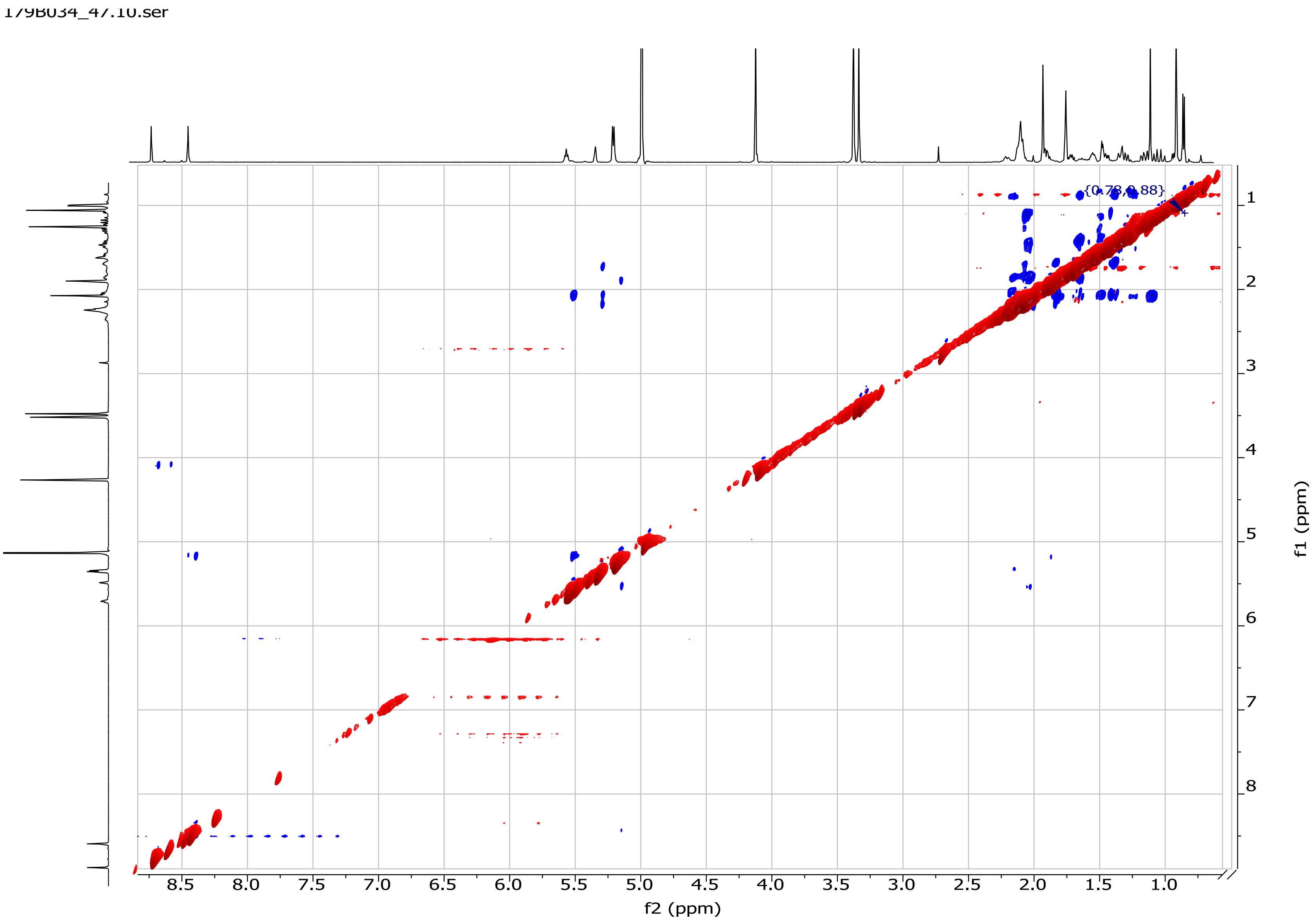


**Figure S23.** NOESY spectrum of Agelasine Y (**3**) in $CD_3OD$.

# Analysis Report

Agilent Trusted Answers

## Sample Information

| | | | |
|---|---|---|---|
| **Name** | 179B034-47 | **Data File Path** | C:\Users\bokeschh\Desktop\QTOF-DATAANALYSIS-ADMIN by HEIDIB\Wei\102919\179B034-47.d |
| **Sample ID** | | **Acq. Time (Local)** | 10/29/2019 8:53:13 AM (UTC-05:00) |
| **Instrument** | Instrument 1 | **Method Path (Acq)** | D:\MassHunter\Methods\FIA_SM_LowFlow.m |
| **MS Type** | QTOF | **Version (Acq SW)** | 6200 series TOF/6500 series Q-TOF B.09.00 (B9044.1 SP1) |
| **Inj. Vol. (ul)** | 1 | **IRM Status** | Some ions missed |
| **Position** | Vial 17 | **Method Path (DA)** | |
| **Plate Pos.** | | **Target Source Path** | |
| **Operator** | | **Result Summary** | |

## Sample Chromatograms

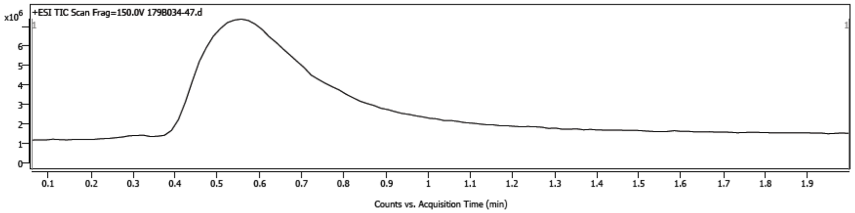


## Sample Spectra

**+ Scan (rt: 0.391-0.888 min)** **C27 H42 N5**

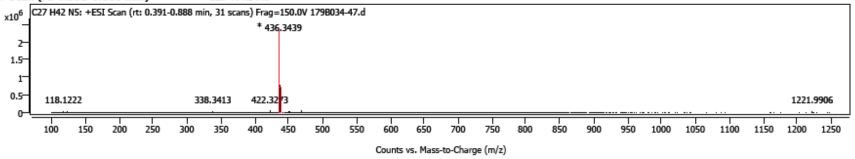


*Spectrum Identification Table*

| Best | ID Source | Name | Formula | Species | m/z | Diff (ppm) | CAS | Score | Score (Lib) | Score (DB) | Score (MFG) | Lib/DB |
|---|---|---|---|---|---|---|---|---|---|---|---|---|
| Yes | MFG | | C27 H42 N5 | M+ | 436.3439 | 0.51 | | 97.80 | | | 97.80 | |

**MassHunter Qual 10.0**
**(End of Report)**



**Figure S24.** HRESIMS spectrum of Agelasine Y (**3**).

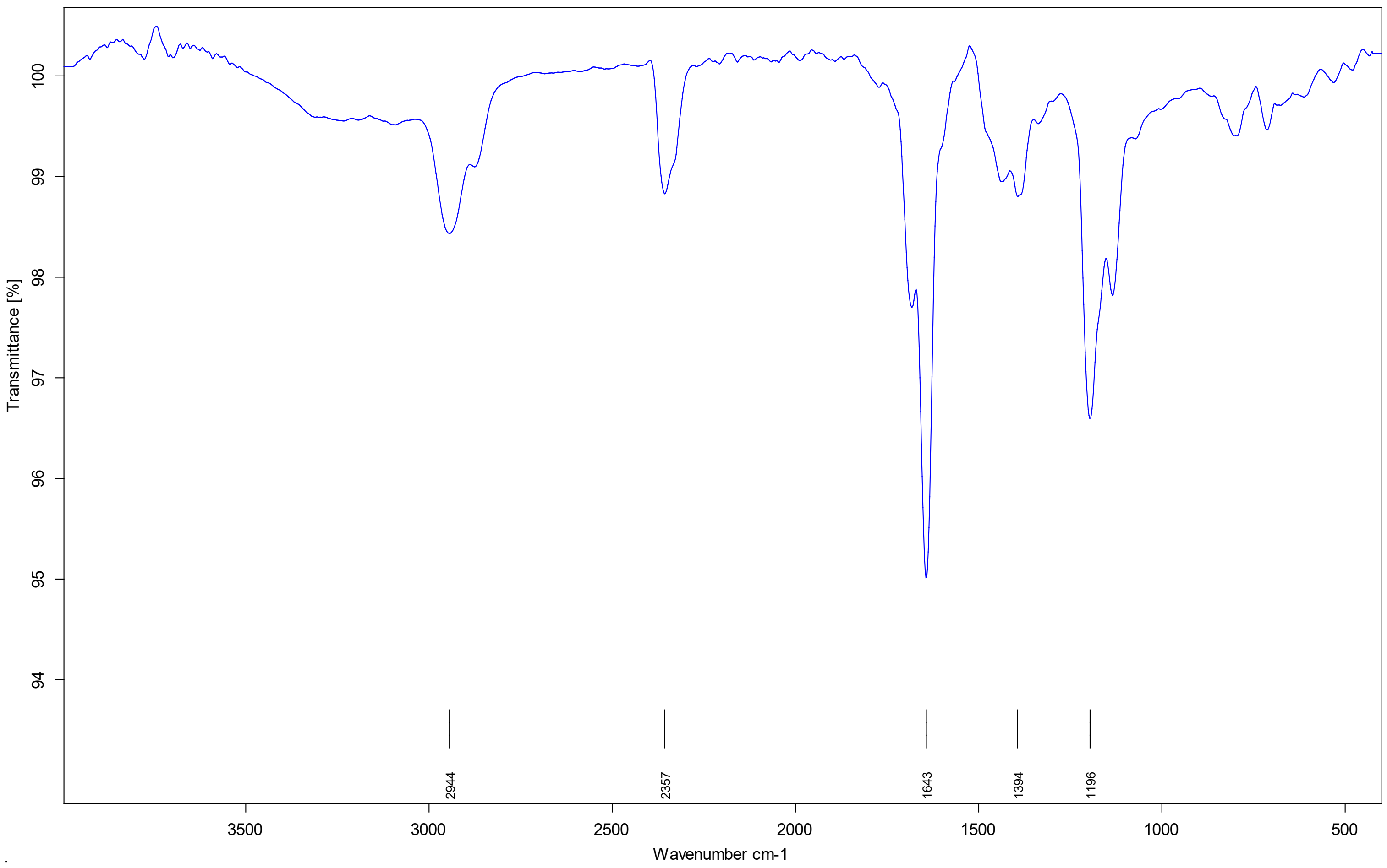


**Figure S25.** IR spectrum (neat) of Agelasine Y (**3**).

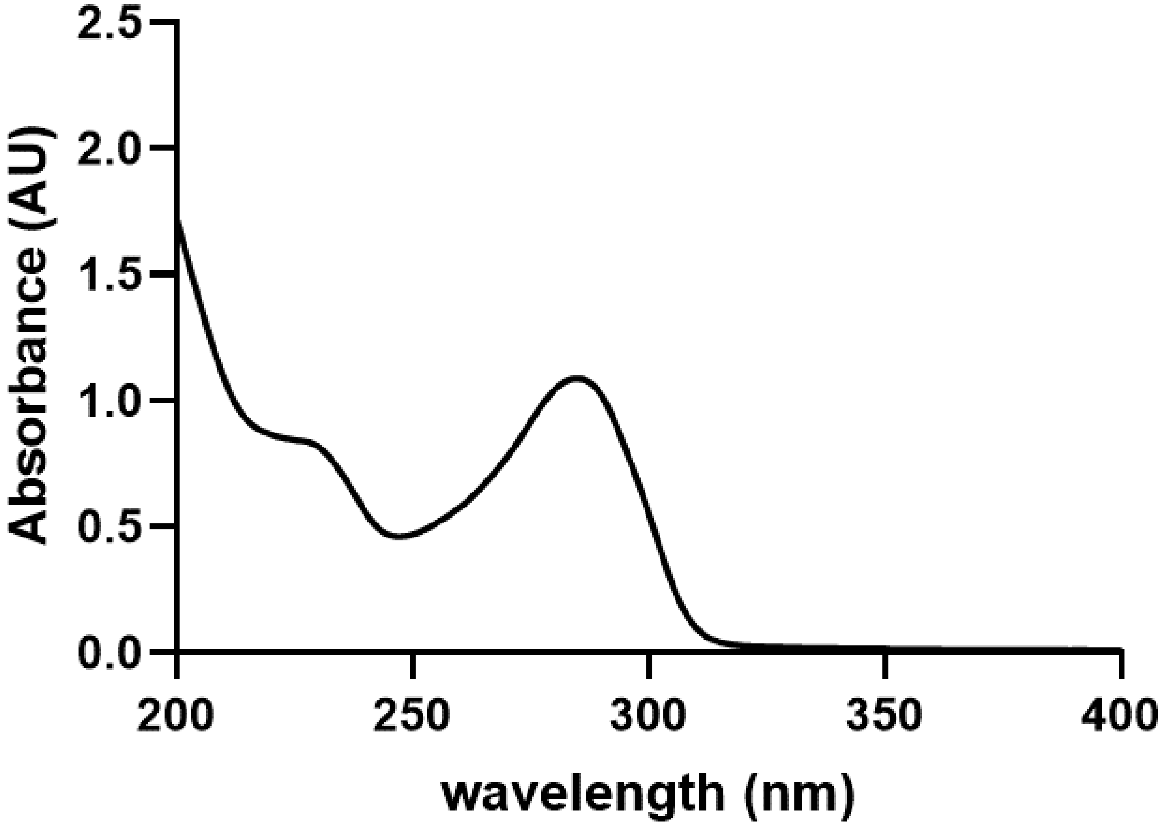


**Figure S26.** UV spectrum of Agelasine Y (**3**).

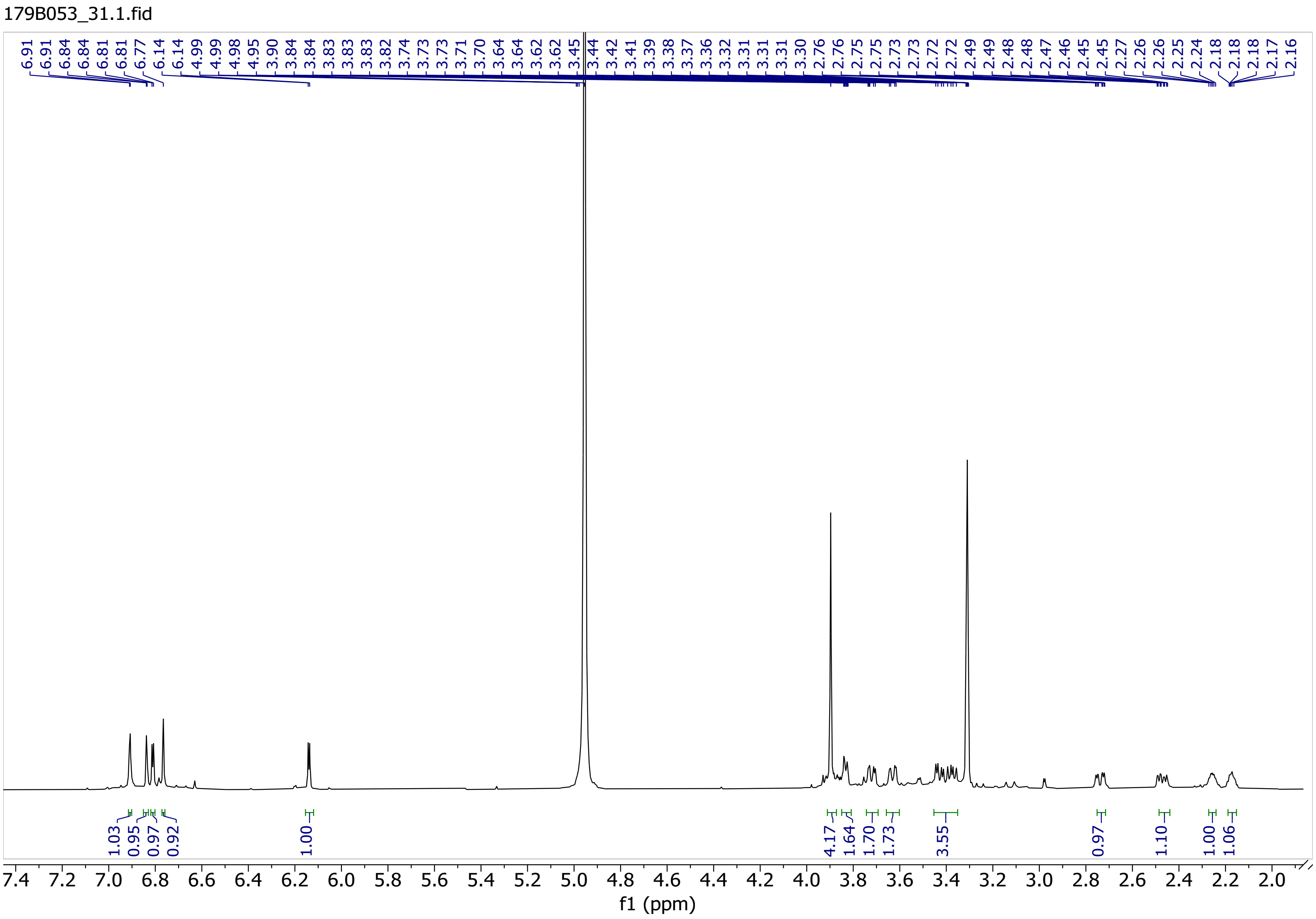


**Figure S27.** $^1$H NMR spectrum (600 MHz) of *N*(1)-methylisoageliferin (**4**) in $CD_3OD$.

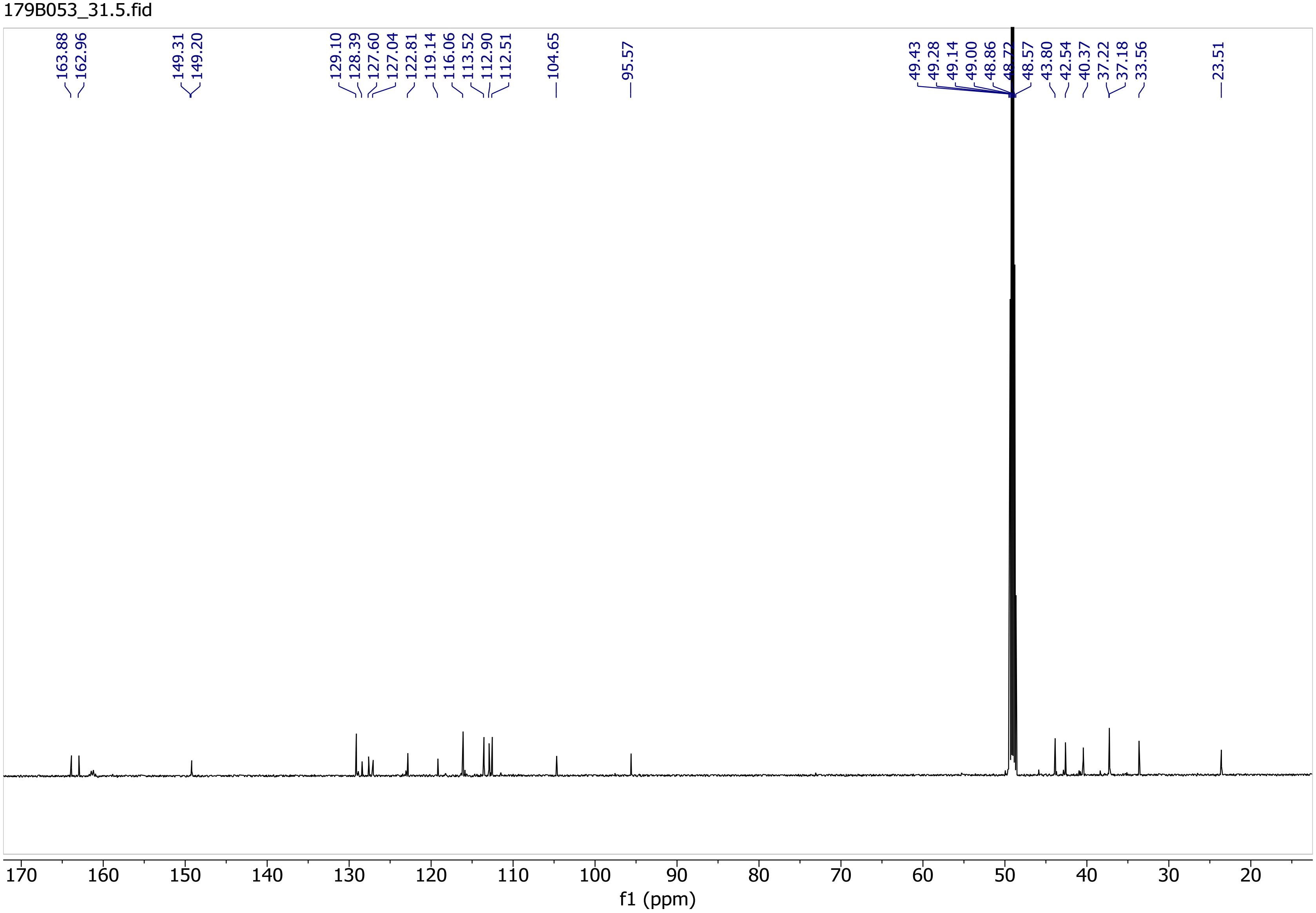


**Figure S28.** $^{13}$C NMR spectrum (150 MHz) of *N*(1)-methylisoageliferin (**4**) in $CD_3OD$.

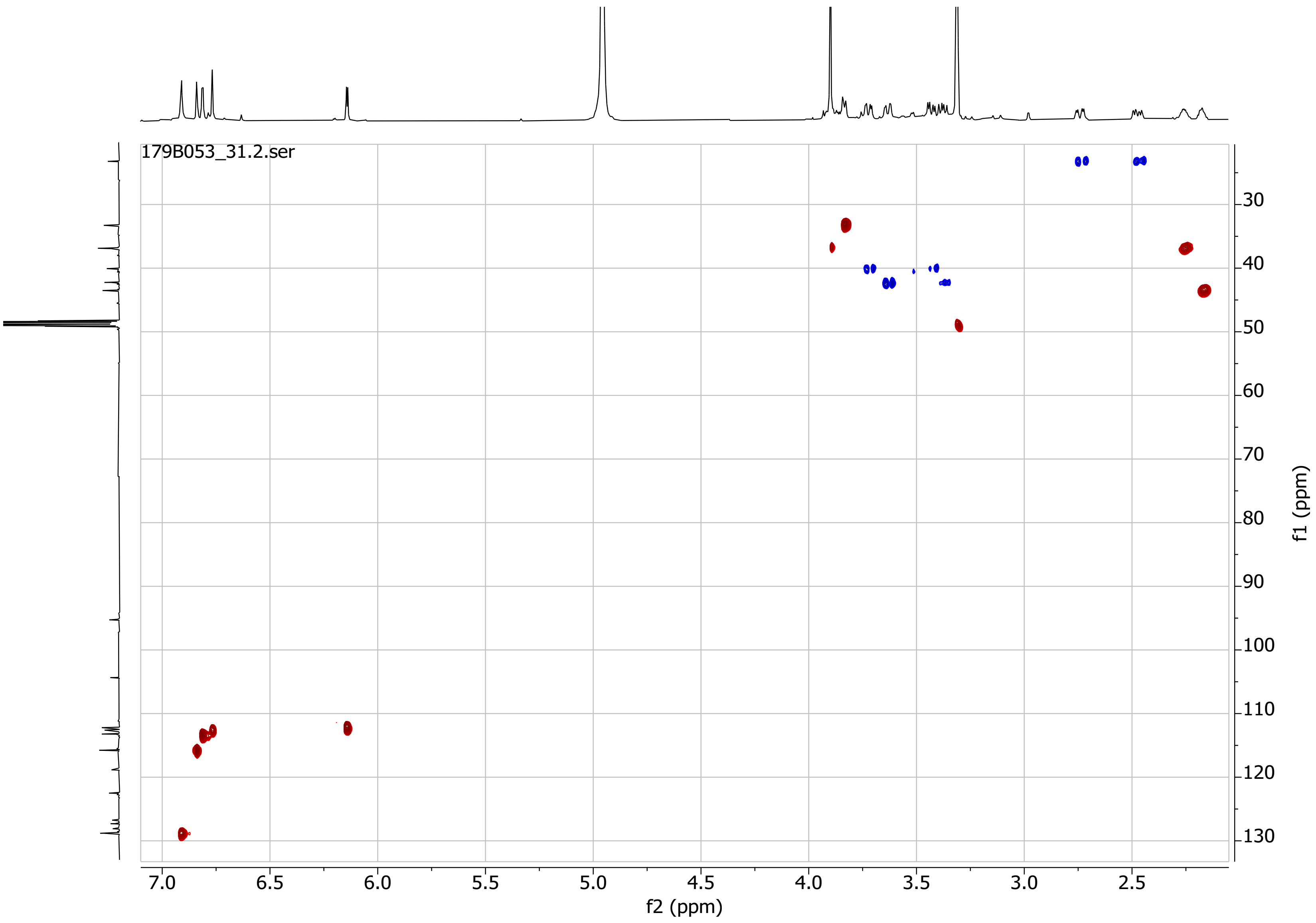


**Figure S29.** HSQC spectrum of *N*(1)-methylisoageliferin (**4**) in $CD_3OD$.

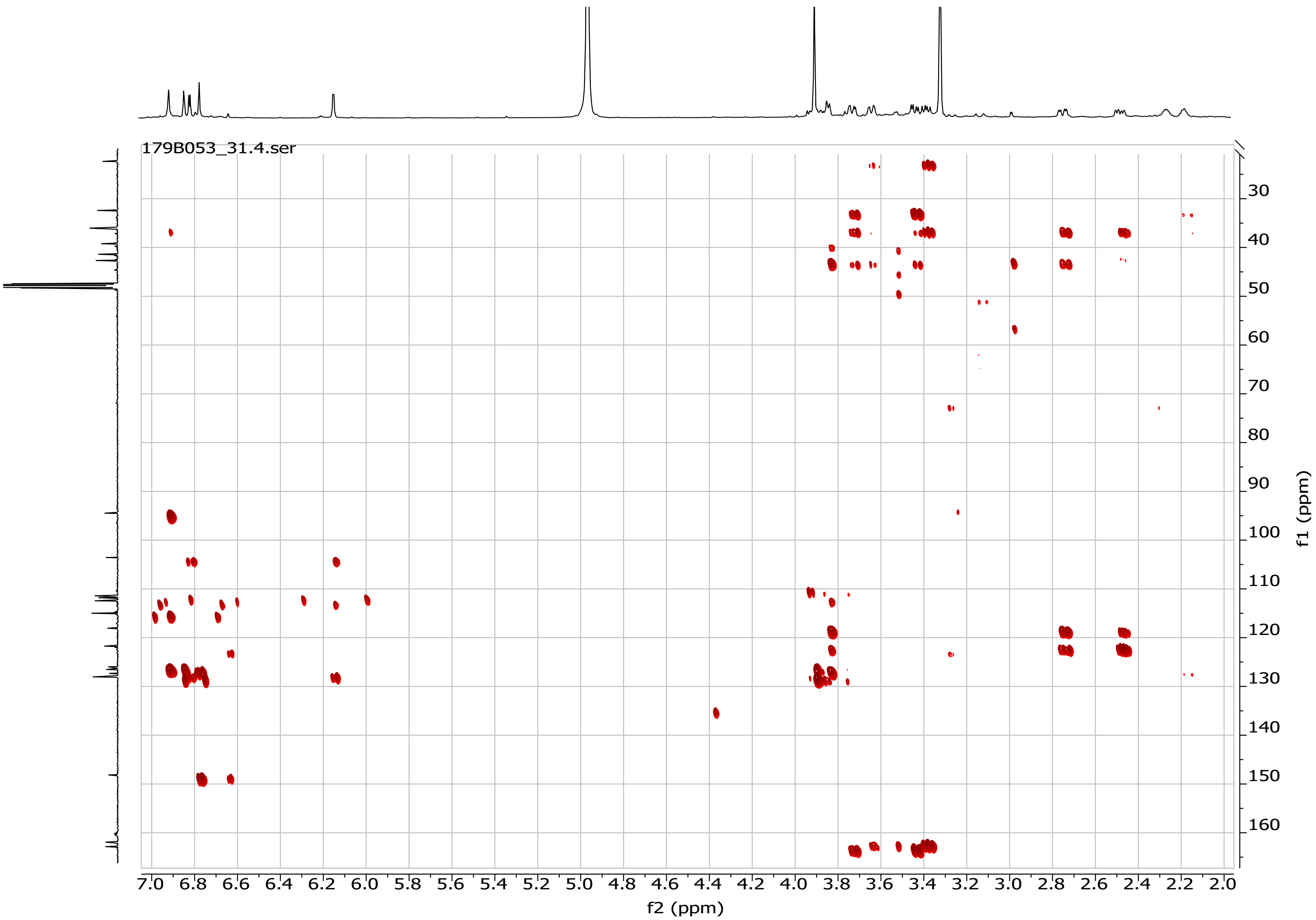


**Figure S30.** HMBC spectrum of *N*(1)-methylisoageliferin (**4**) in $CD_3OD$.

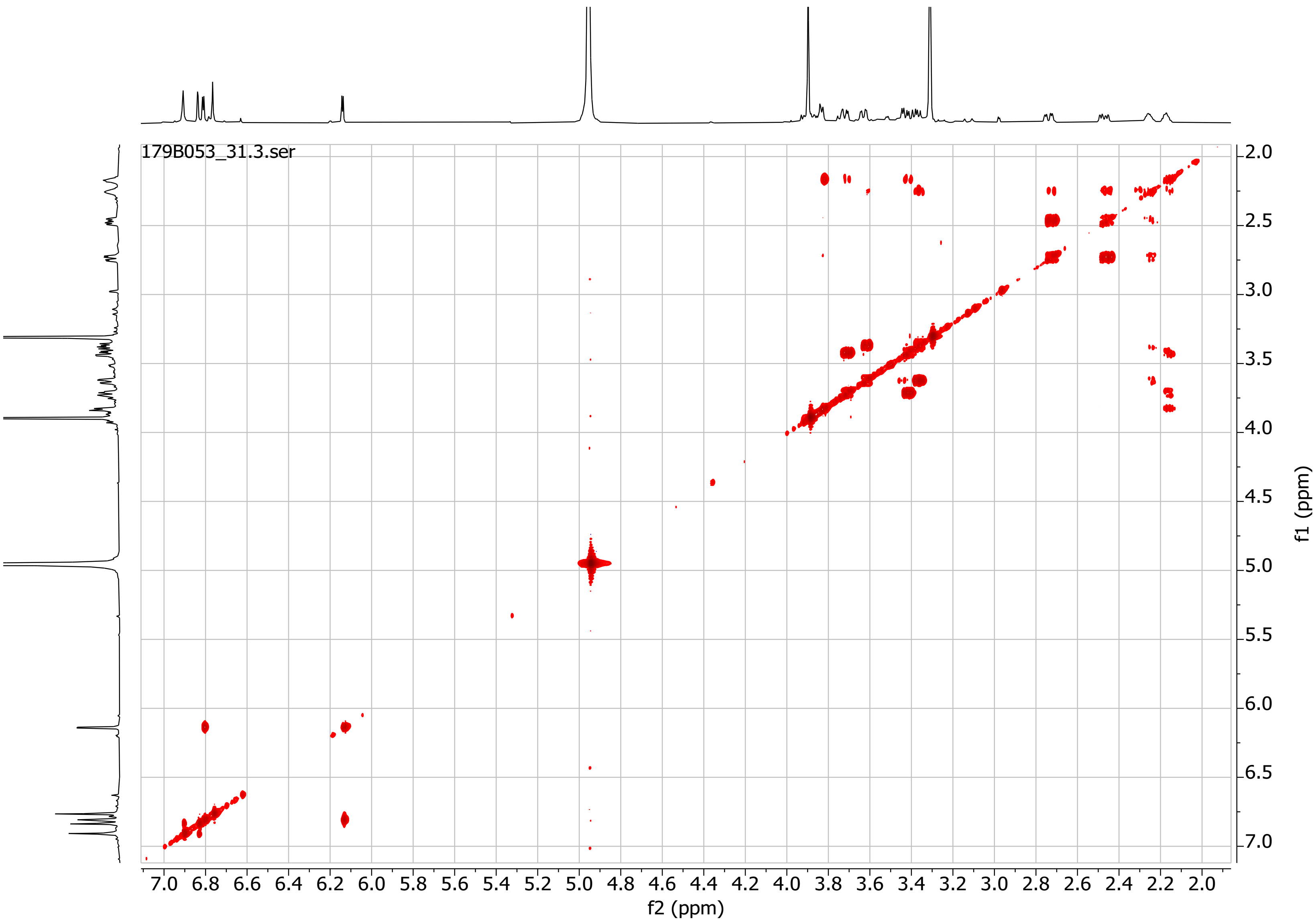


**Figure S31.** COSY spectrum of *N*(1)-methylisoageliferin (**4**) in $CD_3OD$.

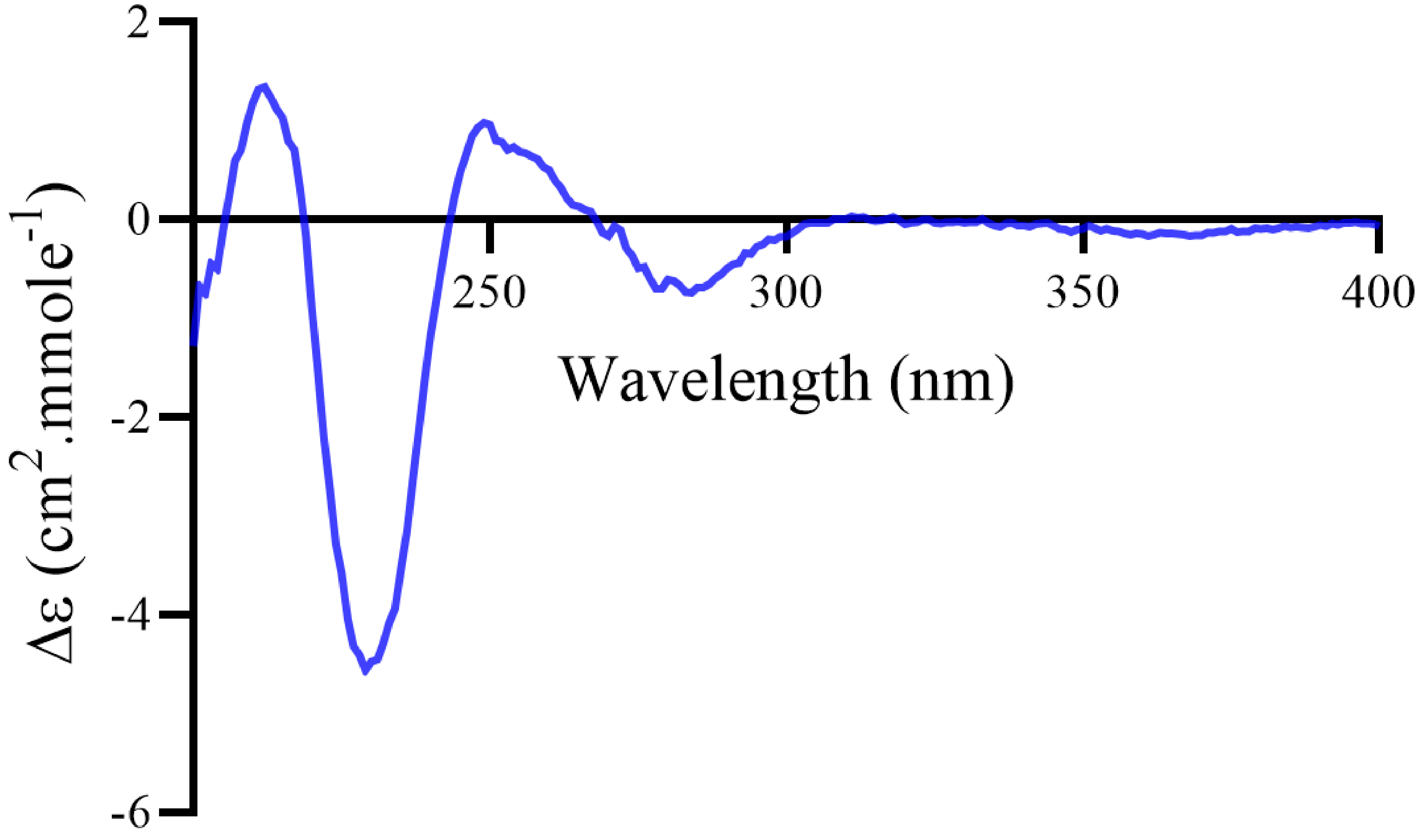


**Figure S32.** ECD spectrum of *N*(1)-methylisoageliferin (**4**).

# Analysis Report

Agilent Trusted Answers

## Sample Information

| | | | |
|---|---|---|---|
| **Name** | 179B053-31 | **Data File Path** | C:\Users\Public\Desktop\QTOF data\Data\Wei\179B053-31.d |
| **Sample ID** | | **Acq. Time (Local)** | 2/6/2020 10:09:41 AM (UTC-05:00) |
| **Instrument** | Instrument 1 | **Method Path (Acq)** | C:\Users\admin\Desktop\methods\FIA_SM_LowFlow.m |
| **MS Type** | QTOF | **Version (Acq SW)** | 6200 series TOF/6500 series Q-TOF B.09.00 (B9044.1 SP1) |
| **Inj. Vol. (ul)** | 1 | **IRM Status** | Success |
| **Position** | Vial 1 | **Method Path (DA)** | C:\Users\Public\Desktop\QTOF data\Data\Wei\179B053-31.d\Results\Qual\Version4\Default.m |
| **Plate Pos.** | | **Target Source Path** | |
| **Operator** | | **Result Summary** | |

## Sample Chromatograms

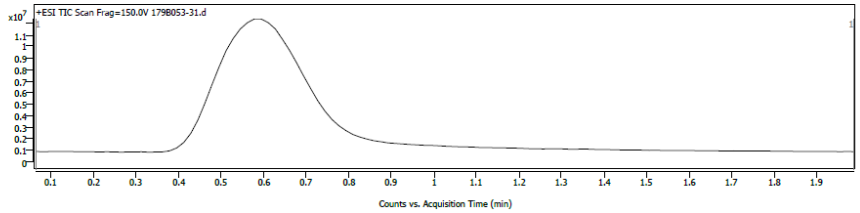


## Sample Spectra

**+ Scan (rt: 0.396-0.827 min) C23 H26 Br2 N10 O2**

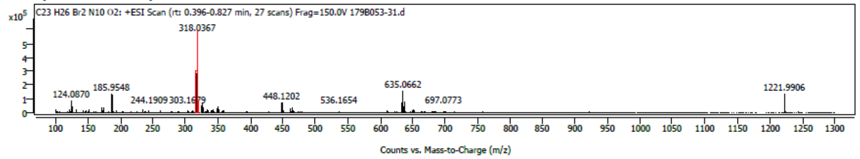


*Spectrum Identification Table*

| Best | ID Source | Name | Formula | Species | m/z | Diff (ppm) | CAS | Score | Score (Lib) | Score (DB) | Score (MFG) | Lib/DB |
|---|---|---|---|---|---|---|---|---|---|---|---|---|
| Yes | MFG | | C23 H26 Br2 N10 O2 | (M+2H)+2 | 317.0374 | -0.52 | | 99.63 | | | 99.63 | |

**MassHunter Qual 10.0**
**(End of Report)**



**Figure S33.** HRESIMS spectrum of *N*(1)-methylisoageliferin (**4**).

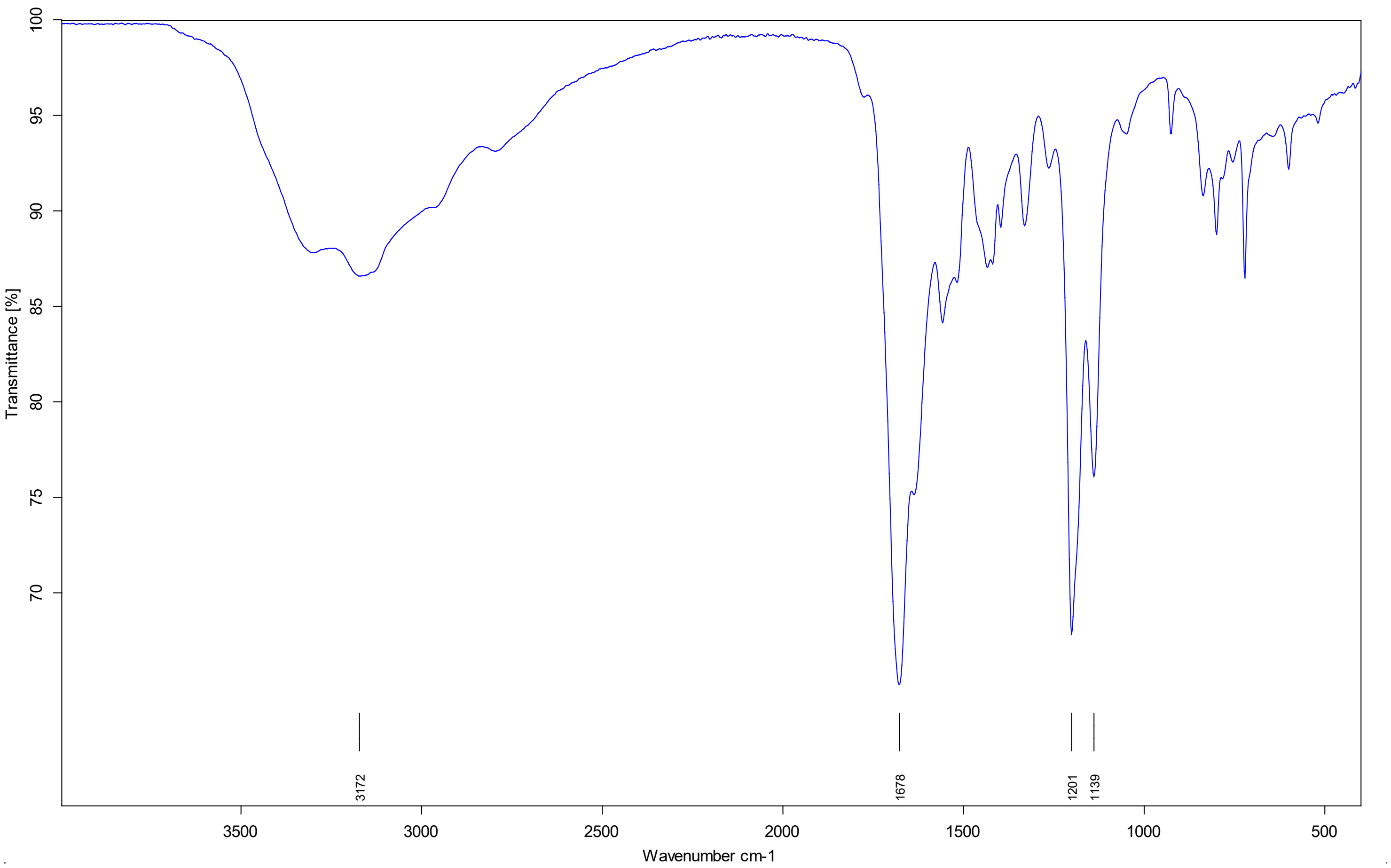


**Figure S34.** IR spectrum (neat) of *N*(1)-methylisoageliferin (**4**).

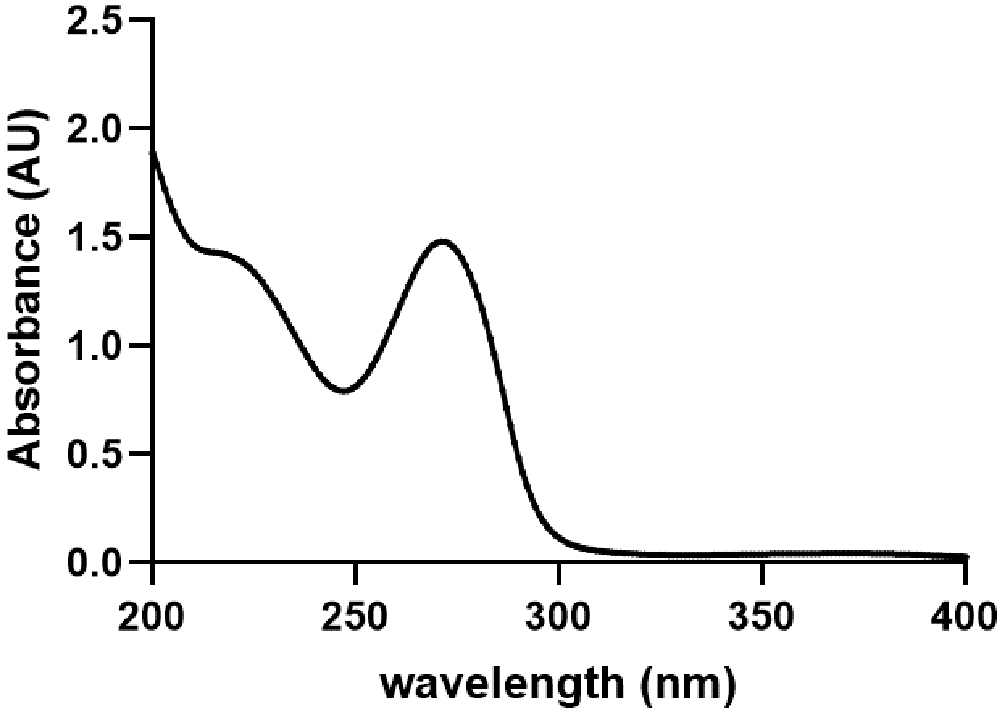


**Figure S35.** UV spectrum of *N*(1)-methylisoageliferin (**4**).

**Table 1** $^{1}$H NMR (600 MHz) data for compounds **5-10** in $CD_3OD$ (*J* in Hz).

| position | 5 | 6 | 7 | 8 | 9 | 10 |
|---|---|---|---|---|---|---|
| 2 | 6.97, d (1.5) | 6.95, d (1.5) | 6.93, d (1.5) | 6.92, d (1.5) | | 6.97, d (1.5) |
| 2’ | | | | 6.92, d (1.5) | 6.91, d (1.5) | |
| 3’ | 6.22, d (4.0) | 6.93, d (1.5) | 6.21, d (4.0) | | | |
| 4 | 7.25, d (1.5) | 7.26, d (1.5) | 7.15, d (1.5) | 7.12, d (1.5) | 7.30, s | 7.22, d (1.5) |
| 4’ | 6.85, d (4.0) | 6.81, d (1.5) | 6.86, d (4.0) | 6.87, d (1.5) | 6.81, d (1.5) | 6.94, s |
| 8a | 3.94, dd (14.8, 3.2) | 3.95, dd (14.8, 3.2) | 3.93, dd (14.8, 3.2) | 3.97, dd (14.8, 3.2) | 3.96, dd (14.8, 3.2) | 3.95, dd (14.8, 3.2) |
| 8b | 3.47, dd (14.8, 4.3) | 3.47, dd (14.8, 4.3) | 3.43, dd (14.8, 4.3) | 3.39, dd (14.8, 4.3) | 3.47, dd (14.8, 4.3) | 3.48, dd (14.8, 4.3) |
| 8’a | 3.87, dd (14.0, 2.7) | 3.86, dd (14.0, 2.7) | 3.86, dd (14.0, 2.7) | 3.89, dd (14.0, 2.7) | 3.87, dd (14.0, 2.7) | 3.86, dd (14.0, 2.7) |
| 8’b | 3.07, dd (14.0, 9.6) | 3.02, dd (14.0, 9.6) | 3.06, dd (14.0, 9.6) | 3.07, dd (14.0, 9.6) | 3.02, dd (14.0, 9.6) | 3.07, dd (14.0, 9.6) |
| 9 | 2.03, m | 1.99, m | 2.04, m | 2.03, m | 2.00, m | 2.02, m |
| 9’ | 2.19, m | 2.18, m | 2.19, m | 2.17, m | 2.18, m | 2.20, m |
| 10 | 3.76, br d (8.5) | 3.73, br d (8.5) | 3.76, br d (8.5) | 3.77, br d (8.5) | 3.74, br d (8.5) | 3.76, br d (8.5) |
| 10’a | 2.85, dd (16.3, 5.3) | 2.84, dd (16.3, 5.3) | 2.83, dd (16.3, 5.3) | 2.82, dd (16.3, 5.3) | 2.85, dd (16.3, 5.3) | 2.85, dd (16.3, 5.3) |
| 10’b | 2.45, ddd (16.3, 9.0, 2.9) | 2.42, ddd (16.3, 9.0, 2.9) | 2.44, ddd (16.3, 9.0, 2.9) | 2.44, ddd (16.3, 9.0, 2.9) | 2.43, ddd (16.3, 9.0, 2.9) | 2.43, ddd (16.3, 9.0, 2.9) |
| 15 | 6.85, br s | 6.85, br s | 6.84, br s | 6.85, br s | 6.87, br s | 6.85, br s |
| NMe | | | 3.91, s | 3.93, s | | |
| N’Me | 3.99, s | 3.97, s | 3.96, s | 3.95, s | 3.98, s | 4.03, s |

**Table S2.** $^{13}C$ NMR (150 MHz) data for compounds **5-10** in $CD_3OD$.

| position | 5 | 6 | 7 | 8 | 9 | 10 |
|---|---|---|---|---|---|---|
| 2 | 123.2, CH | 123.2, CH | 129.1, CH | 129.0, CH | 106.4, C | 123.2, CH |
| 2’ | 111.2, C | 129.0, CH | 111.2, C | 129.0, CH | 129.1, CH | 112.9, C |
| 3 | 97.6, C | 97.6, C | 95.5, C | 95.4, C | 100.0, C | 97.6, C |
| 3’ | 111.6, CH | 95.5, C | 111.5, CH | 95.4, C | 95.5, C | 99.1, C |
| 4 | 114.3, CH | 114.3, CH | 116.4, CH | 116.3, CH | 115.2, CH | 114.2, CH |
| 4’ | 114.7, CH | 115.8, CH | 114.6, CH | 115.8, CH | 115.8, CH | 116.0, CH |
| 5 | 127.1, C | 127.2, C | 127.0, C | 127.0, C | 128.4, C | 127.3, C |
| 5’ | 127.9, C | 127.2, C | 127.9, C | 127.0, C | 127.0, C | 128.5, C |
| 6 | 163.3, C | 163.3, C | 163.9, C | 163.5, C | 162.5, C | 163.3, C |
| 6’ | 164.0, C | 163.6, C | 163.9, C | 163.8, C | 163.6, C | 163.0, C |
| 8 | 38.9, $CH_2$ | 38.8, $CH_2$ | 38.9, $CH_2$ | 39.1, $CH_2$ | 38.8, $CH_2$ | 39.0, $CH_2$ |
| 8’ | 43.0, $CH_2$ | 43.0, $CH_2$ | 42.9, $CH_2$ | 42.8, $CH_2$ | 43.0, $CH_2$ | 43.0, $CH_2$ |
| 9 | 44.2, CH | 44.3, CH | 44.2, CH | 43.9, CH | 44.2, CH | 44.2, CH |
| 9’ | 37.5, CH | 37.5, CH | 37.5, CH | 37.6, CH | 37.5, CH | 37.4, CH |
| 10 | 33.4, CH | 33.3, CH | 33.5, CH | 33.7, CH | 33.4, CH | 33.3, CH |
| 10’ | 24.9, $CH_2$ | 24.9, $CH_2$ | 24.9, $CH_2$ | 25.0, $CH_2$ | 25.0, $CH_2$ | 24.8, $CH_2$ |
| 11 | 127.2, C | 127.0, C | 127.3, C | 127.0, C | 127.1, C | 127.1, C |
| 11’ | 123.0, C | 123.0, C | 123.0, C | 122.9, C | 123.0, C | 123.0, C |
| 13 | 149.2, C | 149.2, C | 149.2, C | 149.3, C | 149.2, C | 149.2, C |
| 13’ | 149.1, C | 149.1, C | 149.1, C | 149.1, C | 149.1, C | 149.1, C |

| | | | | | | |
|---|---|---|---|---|---|---|
| 15 | 113.3, CH | 113.4, CH | 113.3, CH | 113.3, CH | 113.5, CH | 113.3, CH |
| 15’ | 119.4, C | 119.5, C | 119.5, C | 119.6, C | 119.5, C | 119.4, C |
| NMe | | | 37.2, $CH_3$ | 37.4, $CH_3$ | | |
| N’Me | 35.3, $CH_3$ | 37.7, $CH_3$ | 35.4, $CH_3$ | 37.7, $CH_3$ | 37.7, $CH_3$ | 36.7, $CH_3$ |

**Table S3.** $^{13}C$ NMR (150 MHz) data for compounds **3** and closely related clerodane diastereomers[1] in $CDCl_3$. [a]

| position | 3 | *neo-cis-cis-* kolavenol | *ent-neo-cis-trans-* kolavenol | *neo-trans-trans-* kolavenol | *neo-trans-cis-* kolavenol |
|---|---|---|---|---|---|
| 1 | 17.62 | 17.74 | 20.01 | 17.86 | 18.25 |
| 2 | 23.92 | 24.06 | 25.87 | 26.89 | 26.89 |
| 3 | 123.05 | 123.13 | 122.41 | 120.25 | 120.43 |
| 4 | 139.72 | 139.89 | 142.16 | 144.62 | 144.55 |
| 5 | 36.78 | 36.90 | 38.77 | 38.34 | 38.17 |
| 6 | 37.66 | 37.78 | 32.32 | 30.22 | 36.82 |
| 7 | 28.63 | 28.80 | 27.25 | 25.61 | 27.49 |
| 8 | 37.27 | 37.39 | 37.50 | 35.15 | 36.24 |
| 9 | 40.07 | 40.10 | 38.59 | 37.47 | 38.58 |
| 10 | 44.49 | 44.66 | 44.63 | 45.19 | 46.40 |
| 11 | 36.28 | 36.50 | 36.03 | 37.86 | 36.71 |
| 12 | 32.87 | 32.73 | 33.42 | 32.75 | 32.81 |
| 13 | 148.55 | 141.11 | 141.35 | 141.33 | 141.02 |
| 14 | 114.27 | 122.85 | 122.67 | 122.70 | 122.73 |
| 15 | 46.08 | 59.51 | 59.48 | 59.50 | 59.48 |
| 16 | 16.69 | 16.53 | 16.60 | 16.59 | 16.55 |
| 17 | 15.83 | 15.93 | 15.38 | 14.86 | 15.99 |
| 18 | 19.73 | 19.73 | 19.35 | 18.07 | 18.01 |
| 19 | 33.00 | 33.08 | 27.71 | 20.61 | 19.94 |

| | | | | | |
|---|---|---|---|---|---|
| 20 | 17.16 | 17.28 | 26.41 | 20.42 | 18.39 |
| 2′ | 146.85 | | | | |
| 3′ N-Me | 36.25 | | | | |
| 4′ | 148.41 | | | | |
| 5′ | 111.97 | | | | |
| 6′ | 152.35 | | | | |
| 8′ | 144.48 | | | | |
| 10′N-Me | 29.06 | | | | |

a. Chemical shifts were calibrated against known chloroform signals ($\delta_C$ 77.0 ppm)

Reference:

(1) Pelot, K. A.; Hagelthorn, D. M.; Hong, Y. J.; Tantillo, D. J.; Zerbe, P. Diterpene synthase-catalyzed biosynthesis of distinct clerodane stereoisomers. *ChemBioChem* **2019**, *20*, 111-117.
